# Complement membrane attack complex is an immunometabolic regulator of NLRP3 activation and IL-18 secretion in human macrophages

**DOI:** 10.3389/fimmu.2022.918551

**Published:** 2022-09-27

**Authors:** Gisela Jimenez-Duran, Joseph Kozole, Rachel Peltier-Heap, Eleanor R. Dickinson, Christopher R. Kwiatkowski, Francesca Zappacosta, Roland S. Annan, Nicholas W. Galwey, Eva-Maria Nichols, Louise K. Modis, Martha Triantafilou, Kathy Triantafilou, Lee M. Booty

**Affiliations:** ^1^ Immunology Network, Immunology Research Unit, GSK, Stevenage, United Kingdom; ^2^ Institute ofInfection and Immunity, Cardiff University, School of Medicine, University Hospital of Wales, Cardiff, United Kingdom; ^3^ Discovery Analytical, Medicinal Science and Technology (MST), GSK, Philadelphia, PA, United States; ^4^ Discovery Analytical, Medicinal Science and Technology (MST), GSK, Stevenage, United Kingdom; ^5^ Research Statistics, Development Biostatistics, GSK, Stevenage, United Kingdom; ^6^ Immunology Research Unit, GSK, Stevenage, United Kingdom

**Keywords:** complement, immunometabolism, NLRP3 inflammasome, mitochondrial dysfunction, macrophage, reactive oxygen species

## Abstract

The complement system is an ancient and critical part of innate immunity. Recent studies have highlighted novel roles of complement beyond lysis of invading pathogens with implications in regulating the innate immune response, as well as contributing to metabolic reprogramming of T-cells, synoviocytes as well as cells in the CNS. These findings hint that complement can be an immunometabolic regulator, but whether this is also the case for the terminal step of the complement pathway, the membrane attack complex (MAC) is not clear. In this study we focused on determining whether MAC is an immunometabolic regulator of the innate immune response in human monocyte-derived macrophages. Here, we uncover previously uncharacterized metabolic changes and mitochondrial dysfunction occurring downstream of MAC deposition. These alterations in glycolytic flux and mitochondrial morphology and function mediate NLRP3 inflammasome activation, pro-inflammatory cytokine release and gasdermin D formation. Together, these data elucidate a novel signalling cascade, with metabolic alterations at its center, in MAC-stimulated human macrophages that drives an inflammatory consequence in an immunologically relevant cell type.

## Introduction

The innate immune system is an ancient surveillance network able to sense microbial invaders as well as aberrations in cell function. No longer viewed as a static and non-specific part of immunity, the innate immune system employs a plethora of specialized pattern recognition receptors (PRR) in order to monitor homeostasis; these include the Toll-like receptors (TLRs), the RIG-like receptors (RLRs), the NOD-like receptors (NLRs), the C-type lectins, and the complement system. The complement system is a large collection of plasma proteins that can be activated in a cascade-like fashion leading to opsonization of pathogens for phagocytosis and the assembly and deposition of the membrane attack complex (MAC) that kills bacteria or infected/damaged cells through disruption of their membrane integrity. Thus, its job is to recognize pathogen- or damage- associated molecular patterns (PAMPs or DAMPs), to tag the cells of interest and to destroy them. Complement can thus be viewed as another sensor of the pattern recognition system of activation that the innate immune system deploys by recognizing motifs and signatures that trigger the cascade of activation (1, 2) and recent studies have also shown that complement is not an exclusively extracellular system with emerging evidence of the existence of intracellular complement system (termed complosome) with novel homeostatic and immunological functions (3).

Complement-mediated control of aspects of immunity have recently become more evident, particularly considering the NLRs. The NLRs are guardians of normal cell function, triggered in response to aberrations of normal homeostasis. Common NLR triggers are loss of membrane integrity (i. e. through pore-forming toxins, viroporins, lysosomal rupture or MAC), changes in cellular homeostasis (i. e. ROS production, Ca^2+^ efflux, K^+^, Na^+^ ions) and loss of mitochondrial membrane potential (4). Complement components therefore control the NLR-based cellular emergency alarm system: it can provide Signal 1 for inflammasome activation by triggering pro-IL-1β production *via* the C5a-C5aR axis (5, 6) as well as being able to trigger signal 2 either *via* C3a-induced ATP release (7) or *via* MAC insertion into the membrane and Ca^2+^ fluxes (8–10). Finally, it can switch off the alarm using C1q and inhibiting cleavage of caspase-1 (11). Although some elements of complement have compelling links to NLR control, the evidence and mechanisms associated with MAC-mediated control of NLRs are less clear.

Immunometabolism refers to the ability of immune cells to alter their fuel sources in order to align with specific immune functions (12). Such alterations in the bioenergetics of immune cells seem to be triggered by PAMPs, such as lipopolysaccharide (LPS) from bacteria, but the question that remains is whether the host’s innate immune system, such as the complement system, can itself modulate the metabolism of immune cells. Emerging evidence has linked complement with this function including where C3a, C3b and C5a have been shown to shift the metabolic profile of immune cells in order to support pro-inflammatory functions (13). C3aR, C5aR1 as well as complement regulator CD46 signalling have been shown to be required for NLRP3 priming as well as effector T-cell function (13–15). Similarly, autocrine C5aR1 signalling has been shown to increase intracellular ROS and drive IL-1β signalling in T-cells (16, 17). Further hints of complement involvement in immunometabolism have come from clinical studies, where alongside MAC, glycolytic metabolites have been found to be upregulated in samples from rheumatoid arthritis patients (18–20). Most recently, complement was found to drive inflammatory priming and metabolic reprogramming of synovial fibroblasts (21). In addition, it is emerging that complement acts as a global immunometabolic regulator, especially in the brain (22, 23). Thus, these initial findings hint that complement components can regulate immunometabolism, but whether this is also the case for the terminal pathway of complement, MAC, is still unknown.

Given that MAC is widely regarded as an inflammatory trigger and such stimuli have been implicated in modulation of immunometabolic response, we investigated the effect of sublytic MAC in human monocyte-derived macrophages (hMDMs) from the perspective of metabolic control of inflammation. We observed that sublytic MAC was able to skew hMDMs towards a pro-inflammatory glycolytic phenotype. These alterations were able to shift the metabolic phenotype and mitochondrial behaviour to influence pro-inflammatory output *via* NLRP3. These findings further our knowledge of the link between complement and metabolism and have implications for novel approaches in target discovery where aberrant complement activation contributes to disease.

## Results

### Targeted metabolomics downstream of sublytic complement attack highlights enhanced features of glucose-related pathways

The link between metabolism and inflammatory phenotypes of macrophages is well characterised (24–27). These observations have largely relied on the use of LPS as an inflammatory trigger yet understanding endogenous triggers of immunometabolic changes associated with inflammation have more relevance in sterile inflammation scenarios. It is widely accepted that complement is an inflammatory trigger and complement attack on nucleated cells is known to trigger a wide range of effects (28–31). Here, we assessed the consequences of MAC binding to human macrophages with a focus on immunometabolic changes associated with enhancing pro-inflammatory phenotypes that has relevance in sterile inflammation with MAC as an endogenous trigger.

**Figure 1:**
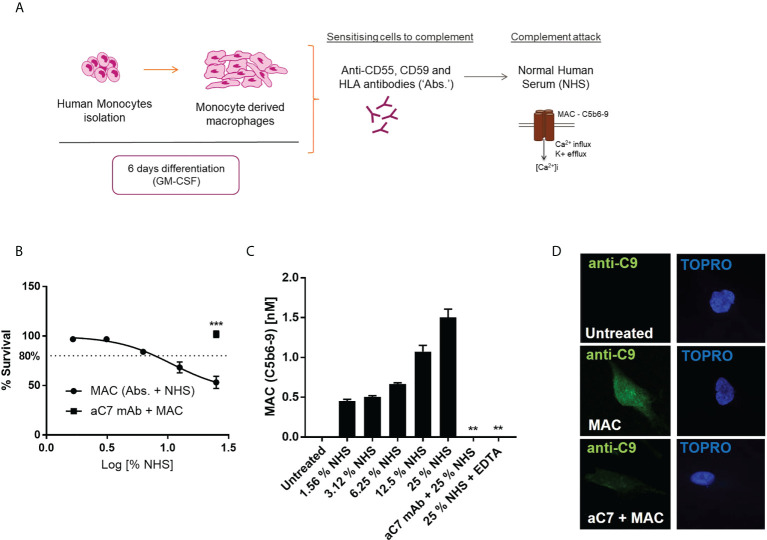
Establishment of suitable sublytic MAC deposition assay in hMDMs. **(A)** schematic of sublytic MAC deposition assay in hMDMs. **(B–D)** hMDMs treated with antibodies (anti-CD55, CD59, HLA) and increasing concentrations of NHS for 1 hour before viability measurement by CellTitreGlo (n=6; B), terminal complement component (TCC) MSD assay (n=3; C) or immunocytochemistry against C9 protein [n=3, representative image shown; **(D)**]. Negative controls were performed by addition of anti-C7 (aC7) + MAC **(B, D)** and EDTA, which blocks complement activation (C only). Error bars represent mean ± S. E. M. ** = p<0.01, *** = p<0.001.

In order to deposit MAC on the surface of hMDMs, we utilized complement regulator antibodies to probe both normal human serum (NHS)-mediated MAC deposition or purified complement protein driven MAC deposition based on previous studies (8, 9, 32, 33) (**Figure 1A**). To ascertain a sublytic dose of NHS corresponding to 80% viability, as has been previously described (34, 35), dose-dependent anti-C7 sensitive viability curves were constructed using both the ATP-dependent CellTitre-Glo (**Figure 1B**) and ATP-independent calcein stain (**Supplementary Figure S1A**), to ensure assay validity during modulations of metabolic function. MAC deposition on hMDMs was confirmed by quantitative measurement of C5b6-9 presence by a Meso Scale Discovery (MSD) based assay (**Figure 1C**), as well as immunofluorescence based detection of C9 (**Figure 1D**), both methods highlighting the suitability of the anti-C7 control. In addition, sublytic levels of MAC were maintained over both 4 and 24 h with maintenance of viability between 70% and 80% (**Supplementary Figures S1B, C**).

**Figure 2:**
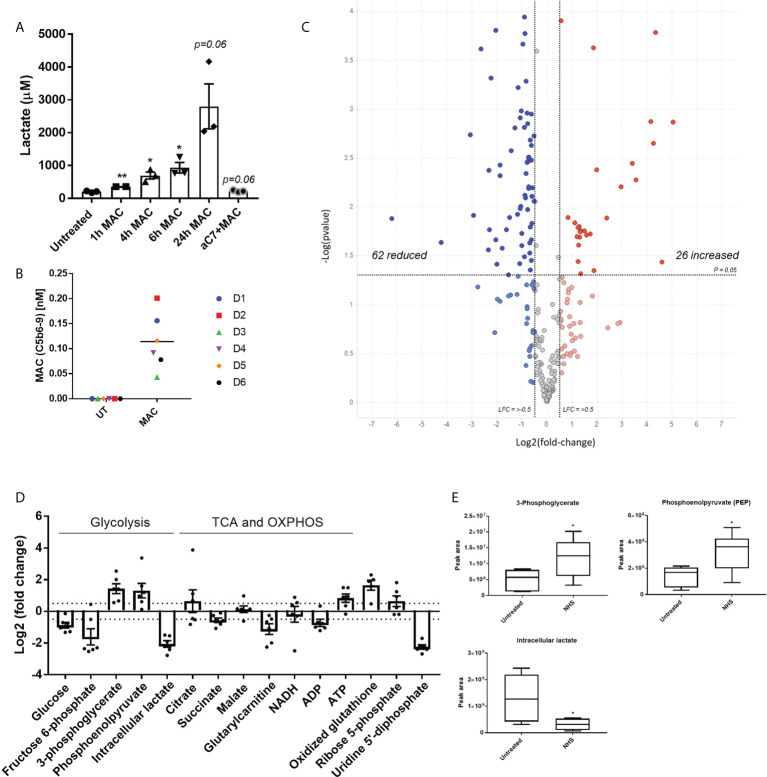
Targeted metabolomics shows alteration of glycolysis intermediates and other glucose related pathways downstream of sublytic complement attack on hMDMs. **(A)** Extracellular lactate produced by hMDMs upon MAC stimulation over time with representative anti-C7 control at 24 h (n=3). **(B)** Quantification of MAC deposition by TCC MSD assay after 4 hours of sublytic complement stimulation with NHS (n=6). **(C)** Volcano plot of targeted metabolomics between untreated and NHS conditions using fold change and p value cut-off at 0. 05 (n=6). **(D)** Fold change of selected metabolites of glucose metabolic pathways between untreated and NHS conditions (n=6). **(E)** Raw values of peak area from selected metabolites (n=6). Error bars represent mean ± S. E. M and whiskers in **(E)** represent min to max. Statistical significance in A and E was determined by unpaired student’s T-test with Welch’s correction for unequal SDs. * = p<0.05, ** = p<0.01.

In order to test our hypothesis that sublytic MAC stimulation of hMDMs may mediate metabolic and inflammatory changes, we first assessed the presence of lactate, a glycolytic waste product, in the extracellular medium of MAC-stimulated hMDMs over time, which showed an anti-C7 sensitive time-dependent increase post-MAC stimulation (**Figure 2A**). Next, we utilized intracellular targeted metabolomics to assess key metabolites from a range of immunometabolic-relevant pathways. hMDMs from six donors were subjected to 4 h of sublytic complement stimulation at a donor-specific pre-defined dose of MAC that corresponded to 80% viability (**Figure 2B**; **Supplementary Figure S2A**). Intracellular metabolomics uncovered 26 upregulated and 62 downregulated features (**Figure 2C**; **Supplementary Figures 2B, C**). Using *in silico* enrichment analysis (36, 37) to analyze these differentially regulated metabolites we observed upregulation in the glycolysis and gluconeogenesis and other glucose-mediated pathways and downregulation of mitochondrially associated pathways such as fatty acid oxidation (**Supplementary Figure S2C**). Focusing on pathways associated with carbon metabolism that are commonly altered during metabolic response in macrophages, changes in glucose metabolism driven by sublytic complement were observed (**Figures 2D, E**; **Supplementary Figure 2D**) with significant decreases in early glycolysis (glucose, fructose-6-phosphate) and parallel glucose metabolic pathway intermediates (nucleotide sugar metabolism; uridine 5’-diphosphate). We also observed a significant increase in later glycolysis pathway metabolites (3-phosphoglycerate, phosphoenolpyruvate; **Figure 2E**). Intracellular lactate was decreased in hMDMs treated with NHS, suggesting an expedited export of lactate corresponding to data in **Figure 2A** (**Figure 2E**). In addition, an increase in oxidized glutathione implicated cellular oxidative stress whilst a decrease of glutarylcarnitine suggested disruption in mitochondrial fatty acid oxidation, as observed in the downregulated pathway analysis (**Supplementary Figures 2C, D**), as well as depletions in fructose-6-phosphate with increased 3-phosphoglycerate (**Figure 2D**), indicative of enhanced flux through the pentose-phosphate pathways. We did observe a large shift in caffeine metabolism between NHS-treated cells versus control, but this is likely an artefact of adding non diet controlled human serum to cells. Together, these data indicate an enhanced utilization of glucose and build-up of end-stage glycolytic intermediates, with potential implications for branch-pathways such as nucleotide sugar metabolism and the pentose phosphate pathway.

### Proteomics analysis in MAC stimulated hMDMs supports shift in aerobic glycolysis, mitochondrial dysfunction by altered pyruvate metabolism and other altered mitochondrial pathways

**Figure 3:**
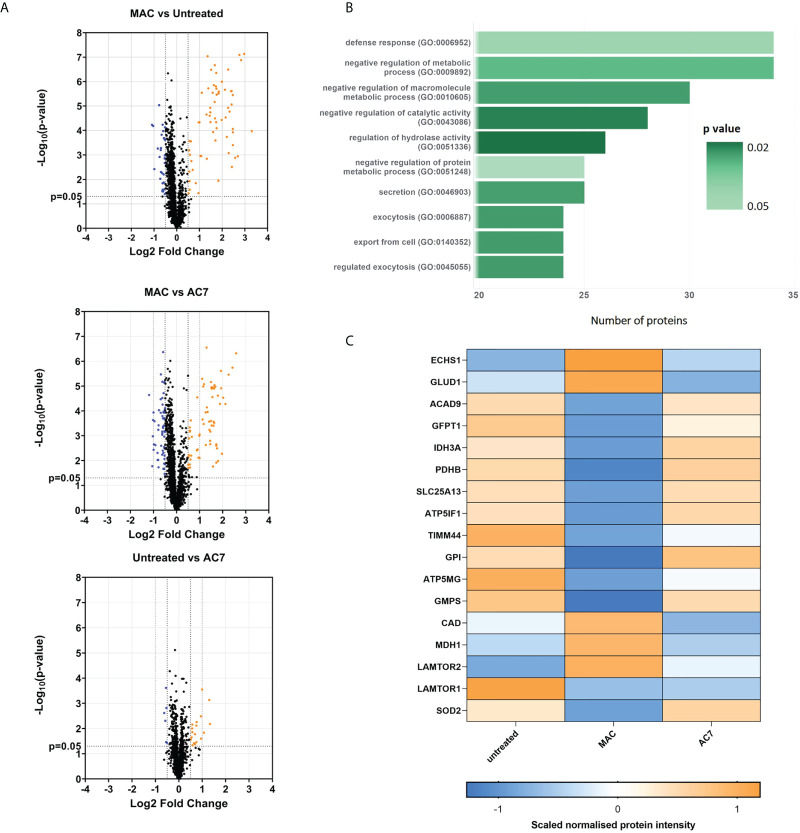
Proteomics analysis shows alteration of glycolysis and mitochondrial proteins and other glucose related pathways downstream of sublytic complement attack on hMDMs. hMDMs treated with sublytic MAC, anti-C7 or untreated controls for 4 hours (n=6 independent donors). **(A)** Volcano plots representing all proteins from the analysis using a fold change cut-off of 0. 5 and unpaired t-test p < 0.05. **(B)** Top 10 list of MAC upregulated pathways from proteomics data using statistical enrichment test for GO biological processes analysis (N= 99 upregulated proteins). See *methods section*. **(C)** Hierarchical clustering of normalized protein intensities (z-score) for significantly regulated metabolic proteins of interest, which were identified as described in the main text and methods section. Statistical analysis performed in **(C)** was one-way ANOVA, FDR corrected, with post-hoc Tukey’s test p < 0.05 significant for both Untreated vs MAC and MAC vs AC7 (N= 17 regulated proteins).

In addition to targeted metabolomics, we proceeded to utilize exploratory proteomics analysis of hMDMs in acute response to sublytic MAC to gain further mechanistic insight into those proteins that immediate alter in response to MAC stimulation. Proteomic analysis of MAC-treated hMDMs uncovered 462 significantly regulated proteins, based on hierarchical clustering of normalized protein intensities (z-score; **Supplementary Figure S3A**, **Supplementary Tables S1****,** **2**), and based on a one-way ANOVA comparing MAC to both untreated and anti-C7 + MAC control groups. Volcano plot analysis of the comparative groups importantly highlights the return to the untreated baseline when anti-C7 was deployed, and the similarity in global changes comparing MAC to the controls (**Figure 3A**). Three distinct clusters emerged from the heatmap analysis (**Supplementary Figure S3A**) corresponding to response to MAC relative to controls (**Supplementary Figure S3B**). Within these clusters, cluster 2 and 3 were modulated by MAC and returned to baseline upon addition of anti-C7, with opposite directionality highlighting their MAC-dependent regulation. Using gene ontology enrichment analysis (38–40) of those differentially regulated proteins upon MAC treatment, we observed the top ten upregulated pathways were involved in the regulation of protein and macromolecular metabolic processes, as well as defense response (**Figure 3B**), while downregulated pathways included small molecule metabolic process, generation of precursor metabolites and energy, and response to external stimulus (**Supplementary Figure S3C**).

We were intrigued to notice a number of significantly MAC-regulated metabolic proteins (**Figure 3C**). Within this group we noticed alterations in early stages of glycolysis (GFTP1, GPI), inhibition of pyruvate dehydrogenase (PDHB) and changes in glutamate and glutamine metabolism (GFPT1, GLUD1, GMPS, CAD). In addition, a number of proteins involved in mitochondrial respiration (notably ATP5GM, ATP5IF1 and IDH3) and metabolite transport (LAMTOR2, SLC25A13) were also altered. These data were supported by longer term transcriptional changes seen in Hypoxia inducible factor alpha (HIF1α), 6-phosphofructo-2-kinase/fructose-2,6-biphosphatase 3 (PFKPB3), IL-1β and PDK4, all of which have been linked to supporting longer-term metabolic alterations in macrophages (41) (12, 42) (**Supplementary Figure 3D**). We did not observe any changes in caffeine metabolism enzymes at a proteomic level. Altogether, this indicative proteomics investigation provides an initial insight into translatable mechanistic effects seen upon MAC stimulation in hMDMs and further suggests the involvement of glycolysis as well as mitochondrial dysfunction in response to sublytic MAC.

### Sublytic MAC stimulation skews hMDM metabolism towards glycolysis

**Figure 4:**
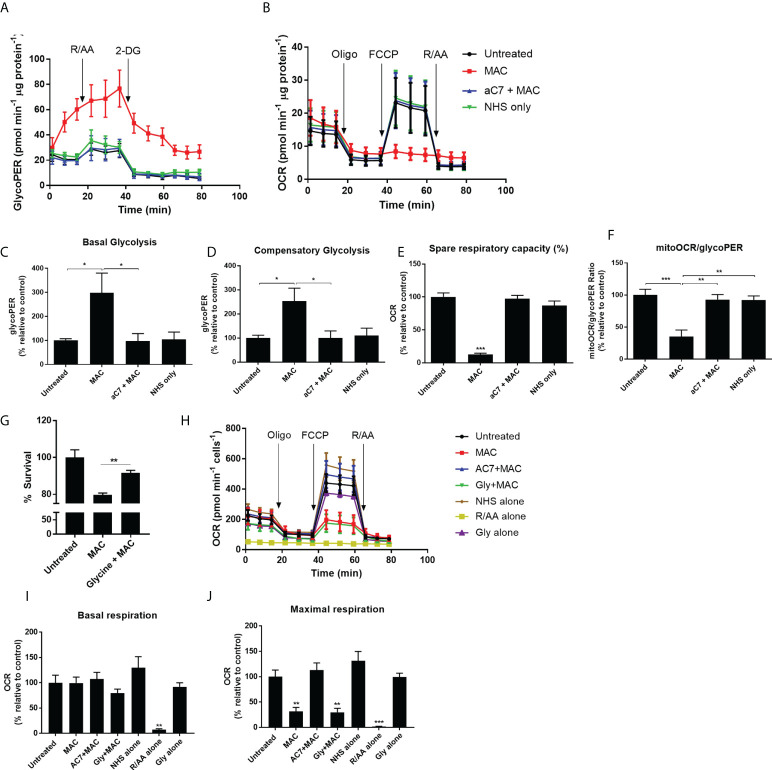
Real-time kinetic metabolic analysis by Seahorse XF technology reveals mitochondrial perturbations and glyolytic upregulation. **(A–F)** hMDMs stimulated with MAC, anti-C7 + MAC or NHS only (no complement sensitising antibodies prior to NHS) before running a Glycolytic Rate Test **(A, C, D)** or Mitochondrial Stress Test **(B, E)** (n=4). Parameters in C-F were calculated using Wave software. **(G)** hMDMs were incubated with 5 mM glycine prior to MAC stimulation for 4 hours and cell viability was measured by CellTitre-Glo. (n=3) **(H–J)** Mitochondrial stress test of hMDMs incubated with MAC or MAC + anti-C7, Glycine + MAC, NHS alone (no antibodies, serum only), Rotenone/Antimycin A (0. 5 uM) or glycine alone (5 mM) for 4 hours prior to initiation of Seahorse assay (n=3). **(H)** kinetic trace; **(I)** basal respiration; **(J)** maximal respiration. Statistical significance between MAC and all the other control groups was assessed by **(G)** unpaired student’s t-test with Welch’s correction for unequal SDs or **(C–F, I, J)** a 1-way ANOVA with post-hoc Tukey’s test. Error bars represent mean ± S. E. M. * = p<0.05, ** = p<0.01, *** = p<0.001.

Since both our metabolomics and proteomics studies indicated enhanced glycolysis as well as mitochondrial dysfunction, to gain further insight into the metabolic alterations in response to sublytic MAC in hMDMs, we utilized Seahorse XF Extracellular Flux Analysis (**Figure 4**). Optimized cell densities of hMDMs (**Supplementary Figure 4A)** were stimulated with sublytic MAC and were metabolically explored using the Glycolytic Rate Test (**Figure 4A**) or the Mitochondrial Stress Test (**Figure 4B**) immediately after MAC addition. hMDMs treated with sublytic MAC exhibited immediate increases in glycolytic metabolism as measured by Glycolytic Proton Efflux Rate (GlycoPER; Figu**re 4A**) as observed in the enhanced basal and compensatory glycolysis (**Figures 4C, D**), yet no change in basal oxygen consumption rate (OCR; **Figure 4B**) and a stark and complete collapse of mitochondrial maximum respiration and spare respiratory capacity (**Figures 4B, E**), contributing to glycolytic tendencies as measured by the MitoOCR/GlycoPER ratio (**Figure 4F**), as well as alterations in other metabolic parameters (**Supplementary Figure 4B**). These observed changes in metabolic phenotype were MAC-dependent, as they were anti-C7 sensitive, and did not occur in the NHS-only negative control, where human serum is added to cells that have not been sensitized to MAC deposition by the regulatory antibodies. In addition, the presence of glycine, which protects against cell death associated with MAC-deposition (**Figure 4G**), was not able to rescue the MAC-induced metabolic phenotype (**Supplementary Figures 4C–E**), indicating these events were not mediated by the presence of dying cells in the external environment. Moreover, the MAC-induced phenotypic changes were consistent at 4 h and 24 h post-MAC stimulation (**Figure 4H**; **Supplementary Figures 4F, G**). The presence of a basal rate and response to oligomycin in MAC-stimulated conditions at all time points are not suggestive of complete mitochondrial collapse or respiratory inhibition post-MAC stimulation, which would behave similarly to rotenone/antimycin A pre-treated cells (**Figures 4H–J**).

### Sublytic MAC stimulation drives mitochondrial dysfunction

**Figure 5:**
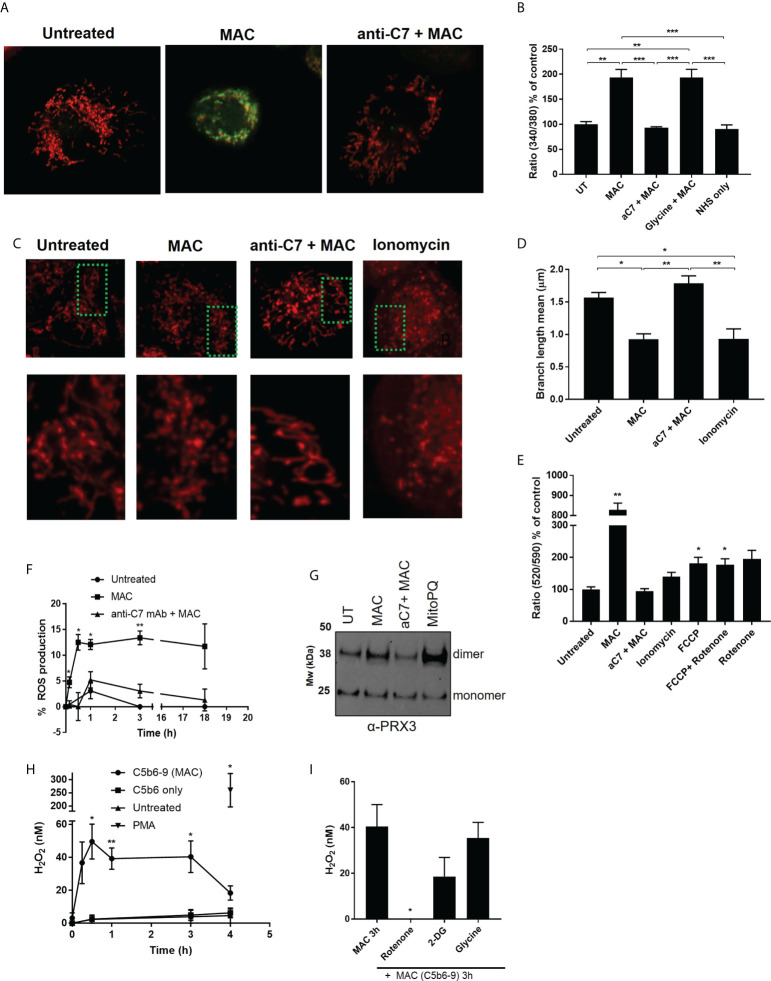
MAC drives disruption of mitochondrial dynamics and membrane potential, triggers increased intracellular calcium and mitochondrial ROS. **(A, B)** Intracellular calcium of hMDMs measured by Fura2 after MAC stimulation (± anti-C7 or glycine (5 mM)), NHS only or untreated for 1 h by fluorescence plate reader (B; n=3) or select conditions by confocal microscopy (A; n=3, representative image shown). **(C)** hMDM were stained with 500 nM MitoTracker Red CMXRos for 15 min before MAC stimulation +/- anti-C7 or 5 μM ionomycin and imaged (n=4 independent donors; representative image shown). **(D)** Quantification of mitochondrial network morphology, expressed as mitochondrial branch length mean, from stimulations described in **(C)** were quantified from confocal microscopy images using MiNa Fiji/ImageJ software (n=4 cells per condition per donor analysed). **(E)** hMDMs were stimulated for 1 h with MAC (+/- anti-C7), 5 μM ionomycin, 1. 2 μM FCCP or 1 μM rotenone and membrane potential measured by JC-10 assay and normalised to untreated cells (n=3). **(F)** Intracellular ROS production measured over time by mean fluorescence intensity using CellROX. hMDMs were stimulated with MAC (+/- anti-C7) and normalised to menadione (30 μM) treatment as positive control (n=3). **(G)** hMDMs were incubated with MAC (+/- anti-C7) or 5 μM MitoPQ for 3 hours and Prx3 dimerization measured by Western blot (n=3; representative blot shown). **(H, I)** H2O2 production of hMDMs stimulated with MAC (anti-CD59 plus C5b6-9 purified components (+/- 50 min pre-incubation with rotenone (0. 5 μM), 2-DG (5 mM) or Glycine (5 mM)) or with C5b6 only or 200 nM PMA only controls (n=4). Statistically significant data between MAC and control groups was assessed by **(B, D)** 1-way ANOVA with post-hoc Tukey’s test and **(E, F, H, I)** unpaired student’s t-test with Welch’s correction for unequal SDs. Significance comparisons in **(E)** are to UT, in **(F, H)** to respective time point of UT control and in **(I)** to MAC treatment. Error bars represent mean +/- S. E. M. * = p<0.05, ** = p<0.01, *** = p<0.001.

As sublytic MAC stimulation of hMDMs mediated increases in glycolytic tendancies and a diminished mitochondrial response to FCCP, we next investigated the effect of sublytic MAC on features of cellular and mitochondrial physiology that determine immunological response (43, 44). Firstly, as calcium influx is the first detectable event in MAC deposition in other cell types (32, 45), leading to mitochondrial depolarization in epithelial cells (9) we also tested calcium flux in hMDMs upon sublytic MAC stimulation. As expected, we were able to observe intracellular calcium flux upon sublytic MAC stimulation as measured by Fura-2 staining (**Figures 5A, B**). As calcium flux can influence mitochondrial morphology and therefore mitochondrial biology and function, we visually assessed the mitochondrial dynamics upon MAC stimulation, and found an anti-C7 sensitive shift towards a fragmented mitochondrial network (**Figure 5C**) which was quantitatively confirmed (46) (**Figure 5D**). In addition, the mitochondrial membrane potential as measured by JC-10 staining was markedly reduced upon MAC stimulation (**Figure 5E**). These results support previous findings in other cell types with regards to calcium flux and membrane potential as well as providing novel evidence that mitochondrial morphology is directly altered as a result of MAC stimulation.

### Mitochondrial ROS production downstream of sublytic MAC

Considering the evidence gathered in response to sublytic MAC stimulation, we hypothesized that the mitochondrial dysfunction may modulate ROS-mediated changes in hMDMs. In response to MAC stimulation, general intracellular ROS as measured by CellROX staining was enhanced (**Figure 5F**), as was dimerization of mitochondrial peroxiredoxin 3, a marker of mitochondrial ROS production (47) to similar levels induced by mitochondrially-targeted superoxide generator MitoPQ (48), (**Figure 5G**; **Supplementary Figure 5A**). In addition, we were able to detect enhanced hydrogen peroxide H_2_O_2_ in the extracellular medium of hMDMs stimulated with purified components of the MAC (**Figure 5H**). Purified components of the MAC were used due to reactivity of the NHS with the H_2_O_2_ assay and these were confirmed to induce sublytic stimulation in both viability and deposition assays (**Supplementary Figures 5B, C**). In addition, this production of H_2_O_2_ was not a result of dying cells, as shown in the addition of glycine, but was significantly sensitive to rotenone, a mitochondrial complex I inhibitor (**Figure 5I**). There was also a slight decrease in H_2_O_2_ detected from cells incubated with the glycolysis inhibitor, 2-DG. Given the rotenone sensitivity and MAC-mediated dimerization of Prx3, we hypothesized that sublytic MAC drives mitochondrial dysfunction and mitochondrial ROS production.

### Sublytic MAC mediated perturbations drive subsequent NLRP3 inflammasome activation in hMDMssa

Immunometabolism refers to the ability of immune cells to alter their fuel sources in order to align with specific immune functions. Our data so far suggested that MAC could modulate such alterations in the bioenergetics of hMDMs promoting glycolysis, which is a fast and responsive means of energy production fueling a pro-inflammatory cytokine response. Inflammasome activation and production of cytokine is often a consequence of such metabolic alterations in the cell, therefore we proceeded to investigate whether stimulation of hMDMs with sublytic MAC was able to induce inflammasome activation.

**Figure 6:**
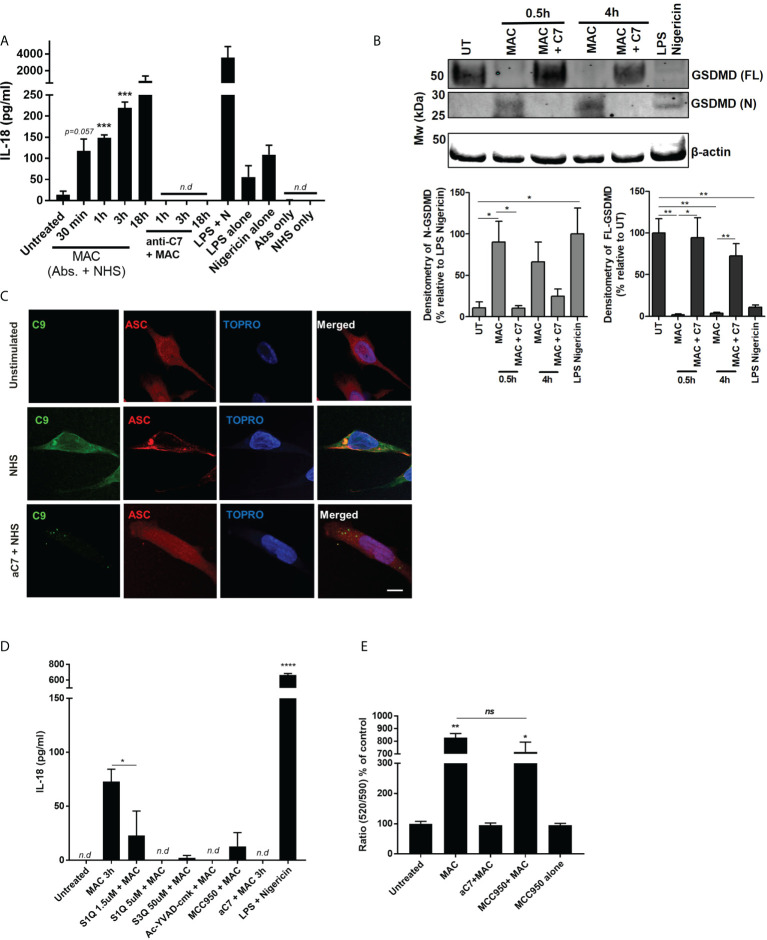
NLRP3 inflammasome activation in hMDMs triggered by MAC. **(A)** IL-18 release by hMDMs detected by IL-18 ELISA of supernatants of hMDMs in 1 million cells per condition (24-well plates) stimulated with MAC or indicated controls for select time points. ‘NHS only’ is serum only, no antibodies, and ‘antibodies only’ is antibodies only, no human serum (n=3) **(B)** Full length and N-terminal GSDMD as assessed by western blot in hMDMs treated with MAC (+/- anti-C7) for 0.5 or 4 hours, as well as LPS-nigericin (3 h LPS (100 ng/mL) + 1 h nigericin (10 µM)). Representative blot image is present alongside densitometry analysis of all three donors using UT/LPS-nigericin controls as relative comparions. **(C)** Immunofluorescence staining against C9 or ASC in cells treated with MAC or MAC + anti-C7 control for 3 hours. Representative images from 1 donor (total, n=3). **(D)** IL-18 ELISA of supernatants from hMDMs in 130k cells per condition (96-well plates) stimulated with MAC for 3 hours with additional inhibitors MCC950 (1 μM), Ac-YVAD-CMK (10 µM), S1QE1. 1 and S3QEL as indicated. Positive control was LPS (100 ng/mL for 3h) followed by nigericin (5 uM for 1 h). **(E)** Mitochondrial membrane potential of hMDMs measured by JC10 after 1h treatment with MAC or controls with MCC950 (1 uM) to block NLRP3 activation (n=3). Statistical analysis performed on **(A, B, E)** was assessed by unpaired student’s t-test with Welch’s correction for unequal SDs. Error bars represent ± S. E. M. * = p<0.05, ** = p<0.01, *** = p<0.001, **** is p = <0.0001. Ns, non significant; Nd, non-detectable.

It was shown that following sublytic MAC stimulation there was a time-dependent release of pro-inflammatory cytokine IL-18 (**Figure 6A**). Release of IL-18 was sublytic MAC dependent, since it was not observed when using NHS alone or antibodies without serum (Abs only), and was sensitive to anti-C7 (**Figure 6A**). In addition to IL-18 release, we were able to detect N-terminal GSDMD (**Figure 6B**), indicative of pyroptotic machinery being activated, as well as ASC speck formation (**Figure 6C**). Using IL-18 release as a proxy for inflammasome formation, we probed the sensitivity of the inflammatory consequences of sublytic MAC stimulation to inhibitors of various stages of the hypothesized signalling pathway (**Figure 6D**; **Supplementary Figure S5B**). As expected, the NLRP3 inhibitor MCC950 and caspase-1 inhibitor, Ac-YVAD-CMK, reduced the IL-18 release.

To test whether the mitochondrial-ROS was responsible for NLRP3 activation and consequential IL-18 release we utilized superoxide production site specific inhibitors of NADH-ubiquinone oxidoreductase, S1QE1. 1, and cytochrome bc1 complex, S3QEL 2 (49). These inhibitors block the superoxide production sites of complex I and III, respectively, without altering oxidative phosphorylation. Interestingly, both of these inhibitors blocked sublytic MAC mediated IL-18 production (**Figure 6D**). In addition, rotenone, as well as the glycolysis inhibitors 2-DG and heptelidic acid, were also able to reduce the IL-18 production (**Supplementary Figure S5D**). To ensure the presence of GSDMD pores were not contributing to mitochondrial dysfunction and collapse we tested the mitochondrial membrane potential after sublytic MAC stimulation in the presence of MCC950, leaving any cellular perturbations to occur independent of NLRP3 activation and resulting GSDMD formation (**Figure 6E**). In the presence of MCC950, mitochondrial membrane potential was similarly affected by sublytic MAC as seen previously, indicating that these changes were not dependent on inflammasome formation and GSDMD pore formation. Overall, we provide evidence that sublytic MAC stimulation in hMDMs can mediate activation of the NLRP3 inflammasome which in turn drives production of IL-18 and GSDMD formation *via* activation of caspase 1. Critically, this initiation of an innate inflammatory process is determined upstream by perturbations in glycolysis, mitochondrial ROS production and mitochondrial dysfunction and contribute to NLRP3 inflammasome formation and downstream inflammatory cytokine release suggesting an axis along which sublytic MAC can signal in hMDMs *via* altering metabolic phenotype and mitochondrial behaviour to increase pro-inflammatory output.

## Discussion

**Figure 7:**
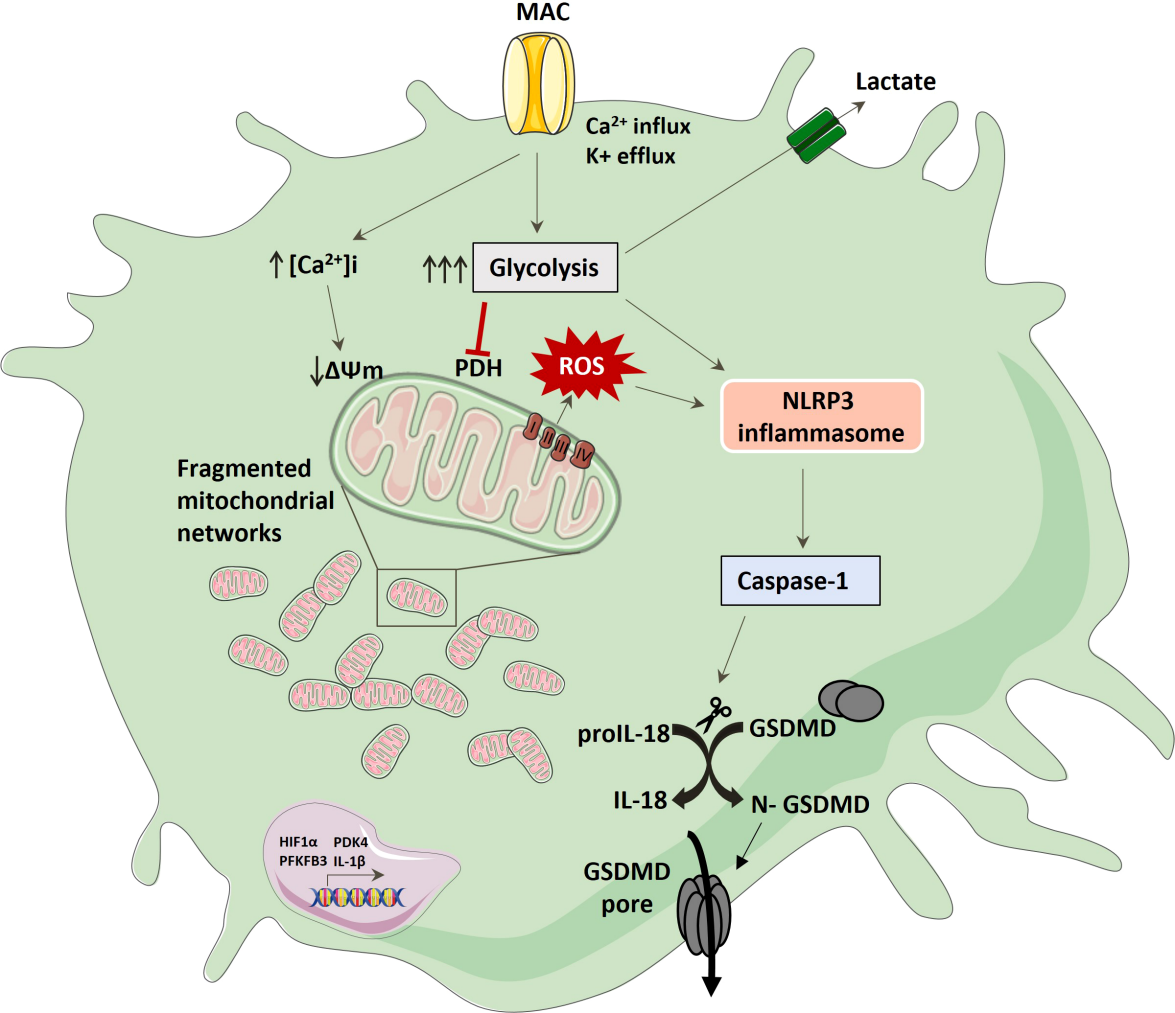
Schematic of hMDM response to MAC stimulation; MAC binding drives glycolysis, ion flux and mitochondrial dysfunction characterised by increased extracellular lactate, fragmented mitochondrial morphology, collapsed mitochondrial respiration and increased mitochondrial ROS production. This activates NLRP3 which in turn mediates caspase 1 activation with subsequent IL-18 and GSDMD cleavage, providing a conduit for active pro-inflammatory cytokine. Longer term changes also include increases in key pathways associated with pro-inflammation.

Immune cells have the ability to alter their metabolic pathways in order to fuel their immune functions. Previous studies have shown that PAMPs, such as LPS can modulate metabolic pathways and skew the phenotype and cytokine output of macrophages, but less is known about endogenous, host-derived regulators of immunometabolism. Complement has recently been emerging as far more than a blood-based anti-microbial detection system and has relevance in multiple autoimmune conditions (50–52). It seems to interact with other innate immune pathways, inflammasomes and metabolic pathways playing a global role as an immunometabolic regulator and seems to function both intra- and extracellularly. Recent studies have shown that different complement components, such as C3a, C3b, and C5a can shift the metabolic profiles of T-cells, synoviocytes as well as cells of the CNS, in the absence of LPS, but whether the terminal complement pathway, MAC, could directly modulate metabolic pathways was unknown. We have previously shown that MAC can trigger inflammasome activation (9) but the question remained how the cell fuels this MAC-dependent inflammasome activation, or whether this occurred in immunological cell types. In this study we provide an insight into how MAC drives a metabolic rewiring in myeloid cells, which primes the cells for inflammatory outputs *via* mitochondrial dysfunction (**Figure 7**).

Through the use of both metabolomic and proteomic analyses we were able to confirm enhanced glucose related pathways as well as heightened oxidative stress and mitochondrial dysfunction in response to MAC. In particular, our results demonstrated downregulation of PDHB, as well as increased gene expression of PDK4, indicating regulation of the pyruvate dehydrogenase complex, supporting aberrant preferential activation of glycolysis (53). It is interesting to mention that PDK4 regulation has also been observed in LPS-stimulated hMDMs in limiting pyruvate import (41) (54). Given this altered pyruvate supply to the mitochondria, modulation of alternative pathways in glutamine, glutamate and nucleotide sugar metabolism through GFPT1, GLUD1 and UDP was observed, aligning to behaviour akin to glycolytic cancer cells that use glutamine/glutamate metabolism to supply the TCA cycle (55, 56). These MAC-induced rewirings of central carbon metabolism was further supported in transcriptional upregulation of HIF1α and PFKFB3, both known for maintenance of pro-inflammatory metabolic phenotypes in macrophages (12, 42, 57). In addition to the glycolysis pathway, TCA cycle and electron transport chain associated proteins were also found to be regulated by MAC and may aid explanation to the molecular mechanism associated with mitochondrial dysfunction. For instance, ATP5IF1, which limits ATP depletion during mitochondrial membrane potential collapse, was reduced in the context of sublytic MAC stimulation, as was SOD2 – a key regulator of mitochondrial superoxide (58, 59). Observations around caffeine metabolism in the targeted metabolomics were likely a result of externally supplied caffeine through obtained samples from non-diet-restricted subjects. No proteomic evidence of changes in caffeine metabolism were observed, restricting this observation to detection in metabolomics only.

In order to confirm the proteomics and metabolomics findings, we proceeded to determine metabolic alterations in hMDMs post MAC stimulation and confirmed a collapse in maximal mitochondrial respiration, enhanced extracellular lactate present and increased glycolysis. In this scenario we suspect that the lactate export is a by-product of a macrophage undergoing a response to MAC, requiring rapid ATP production *via* glycolysis and upregulation of canonical pro-inflammatory response elements such as HIF-1α and IL18. Damage or perforation of the mitochondrial membrane itself is unlikely to explain these phenotypes as oxygen consumption rates prior to FCCP injection (basal mitochondrial metabolism and ATP synthesis) were unaffected post-sublytic MAC stimulation at either time point (**Figure 4**). Instead, the reduction in maximal respiration may be explained by the switch away from pyruvate delivery to the mitochondria, as seen in transcriptional changes as mentioned above. Further investigations into mitochondrial physiology post MAC stimulation uncovered perturbations in mitochondrial dynamics, membrane potential and calcium flux as well as redox biology (**Figure 5**), altogether implicating mitochondrial homeostasis at the heart of the hMDM response to sublytic MAC. These observations of calcium and membrane potential changes are supported by previous findings in lung epithelial and rat mesangial cells (9, 60) and the observations of mitochondrial morphological changes and ROS production provide a novel insight into the downstream response to sublytic MAC stimulation. These MAC-induced observed changes align with previous findings in macrophages stimulated withexternalimmune stimuli such as LPS, including the rotenone-sensitive Complex I driven ROS reaction, (27, 61–63). It is likely, given the sensitivity of the ROS production to complex I inhibitors that reverse electron transport and an altered NADH/NAD+ ratio are contributing to the observed phenotype. This suggests that endogenous triggers, such as MAC, can also alter the phenotype of the immune cells similar to that of well-characterised stimuli such as LPS.

The NLRP3 inflammasome is known to integrate signals derived from ROS, glycolysis, amino acid and lipid metabolism to activate caspase 1 and, consequentially, IL-1β, IL-18 and GSDMD (12, 13). In order to determine whether the MAC-induced metabolic changes observed trigger inflammasome activation, we investigated cytokine release in response to MAC. Interestingly, sublytic MAC stimulation led to release of IL-18, but not IL-1β, as well as ASC speck formation and GSDMD cleavage (**Figure 6**). The absence of secreted IL-1β may be attributed to the lack of a defined inflammasome priming signal, such as LPS, which is dispensable for NLRP3 activation in human monocytes (64). Although C5a stimulation has been shown to act as an NLRP3 priming signal (6, 65) it may not be sufficient to activate pro-IL-1β expression, whilst IL-18 is not limited in this way (64). Therefore, it seems that MAC deposition on cells is inducing a one-step inflammasome activation in hMDMs triggering IL-18 production – bypassing the need of a priming signal. Using IL-18 as a proxy for inflammasome activation we were able to confirm sensitivity of sublytic MAC-induced inflammatory signalling to both MCC950 and Ac-YVAD-CMK, confirming NLRP3 and caspase 1 activation downstream of sublytic MAC stimulation (**Figure 6**). Interestingly, ASC oligomerization co-localized with MAC staining intracellularly (**Figure 6C**), which is in agreement with previous studies where MAC was observed to internalize and co-localize with NLRP3 and ASC (10, 66, 67). It could be speculated that there could be a threshold of MAC activation that favors internalization over endocytosis and/or blebbing (68, 69), with each having distinct functional outcomes. Also interesting, is the concept of inflammasome activation directly from complement components and the requirement for homeostatic disturbances as observed here. Future work should endeavor to address the respective contribution or interplay of direct interactions between complement components and inflammasome machinery, and the requirement of independent drivers arising from signal-driven homeostatic imbalance. In addition, we show that sublytic-MAC mediated IL-18 release from hMDMs is sensitive to inhibitors of mitochondrial ROS production and glycolysis highlighting the need for glycolysis and mitochondrial dysfunction in NLRP3 activation and pro-inflammatory response. The use of the specific superoxide-production site inhibitors of complex I and III, S1QE1. 1 and S3QEL 2, show the requirement of a mitochondrial ROS driven activation of NLRP3, linking together the observed mitochondrial dysfunction and consequential inflammatory response.

The maintained viability in hMDMs post-sublytic MAC stimulation is intriguing, especially considering active inflammasome machinery, yet our data aligns with other studies that observed MAC-driven NLRP3 activation with viability maintenance in M-CSF hMDMs, epithelial cells and dendritic cells (8–10). MAC may drive macrophages into a state of hyperactivation with IL-18 secretion *via* GSDMD without breaching a threshold for complete commitment towards pyroptotic cell death (70) and instead driving IL-18-mediated signalling in neighboring macrophages and other target cells (71, 72). This interesting balance between sublytic MAC signalling and cell death certainly warrants further understanding in the context of innate cell death signals and localized immune responses. In addition, the classical concept that MAC is simply a blood-based anti-microbial pore-forming detection is an oversimplified one when considering complement-host cell interaction. It is believed that MAC lesions cluster within membrane microdomains and are either rapidly shed from the cell membrane or endocytosed – most likely this is dependent on the threshold of MAC clusters on the cell membrane. Our data suggests that upon MAC stimulation, MAC is able to trigger metabolic events that are able to influence and shape the inflammatory response suggesting MAC is an endogenous modulator of immunometabolism. This phenotypic shift can be characterized in the short term by enhanced glycolytic flux and calcium influx, and altered mitochondrial morphology, ROS production and membrane potential, and in the long term by a pro-glycolytic and pro-inflammatory transcriptional response. Interestingly, we do observe some elements of metabolic failure as measured by proton leak at 24 h, suggesting that after a longer time period the cells may undergo irreversible changes in metabolic function, which may lead to cellular dysfunction and failure. This metabolic rewiring could be crucial for inflammatory conditions where there is chronic complement activation, such as RA where sublytic MAC and increased glycolysis can contribute to inflammation (18–20, 73) and diabetes, where high levels of glucose cause glycation of CD59 with subsequent MAC activation (74). In such conditions, MAC-mediated immunometabolically-altered macrophages could be playing a central role in the outcome of the disease and thus MAC or downstream MAC-related consequences could be attractive future therapeutic targets. In addition, future studies could focus on investigating the effect of MAC deposition at sublytic levels in the context of macrophage function, such as phagocytosis or cytokine release, in disease-relevant settings, using models, complex co-cultures or external metabolomic landscapes. This will provide a robust and relevant insight that builds on these *in vitro* findings. We suspect that uncovering specific macrophage polarization and functional effects will in itself become an interesting narrative to follow. Our data provides evidence that MAC goes far beyond its popular image of a simple pore and demonstrates a novel involvement of MAC in the complement-metabolism-inflammasome axis of macrophages specifically (13). Only by increasing our understanding of these new mechanisms and functions of the complement system can we understand its homeostatic functions and guide the comprehensive selection of optimal targets for therapeutic interventions in the future.

**Table:** Reagents List.

REAGENT	SUPPLIER	IDENTIFIER
**Antibodies**		
**Anti-human C7 monoclonal**	Quidel	A221
**Anti-human CD55 monoclonal**	IBGRL	BRIC 216
**Anti-human CD59 monoclonal**	IBGRL	BRIC 229
**Purified anti-human HLA-A,B,C monoclonal**	BioLegend	311402
**IRDye^®^ 800CW Donkey anti-Rabbit IgG Secondary Antibody**	LI-COR	926-32213
**IRDye^®^ 680LT Donkey anti-Mouse IgG Secondary Antibody**	LI-COR	926-68072
**Rabbit Anti-Peroxiredoxin 3**	Abcam	Ab73349
**Rabbit anti-Gasdermin D L60**	Cell Signalling	93709
**Mouse anti-β-actin monoclonal**	Sigma	A2228
**Anti-human C9 neoantigen monoclonal WU13-15**	HyCult	HM2264
**Rabbit anti-human ASC (AL177)**	Adipogen	AG-25B-0006-C100
**Donkey anti-Mouse IgG (H+L) Secondary Antibody, Alexa Fluor^®^ 488 conjugate**	Thermo-Fisher	A-21202
**Donkey anti-rabbit IgG Secondary Antibody, Alexa Fluor^®^ 546 conjugate**	Thermo-Fisher	A10040
**Biotinylated C5b-9**	(Abcam, biotinylated in-house)	ab66768
**Ruthenylated anti-C6**	(Quidel, ruthenylated in-house)	A219
**Chemicals, Peptides, and Recombinant Proteins**		
**LPS Lipopolysaccharides from *E. coli* O55:B5**	Sigma	L2880-10MG
**Carbonyl cyanide 4-(trifluorome-thoxy)phenylhydrazone (FCCP)**	Sigma	C2920-10mg
**Nigericin**	Invitroogen	TLRL-NIG
**Rotenone**	Sigma	R8875
**Glycine**	Sigma	50046
**Methylmethanethiosulfonate (MMTS)**	Sigma	208795
**Sodium dodecyl sulfate (SDS)**	Sigma	L3771
**Sodium deoxycholate**	Sigma	30970
**MitoPQ, mitochondria-targeted redox cycler**	Abcam	ab146819
**MitoTracker™ Red CMXRos**	Thermofisher	M7512
**Fura-2 AM, Ca2+ selective fluorescent indicator**	Abcam	ab120873
**Human recombinant GM-CSF**	R&D Systems	215-GM-010/CF
**Ionomycin**	Sigma	I3909
**2-deoxyglucose**	Sigma	D6134- 1G
**Heptalidic Acid**	Abcam	ab144269
**MCC950**	Sigma Life Science	CP-456773
**AC-YVAD-CMK**	*In vivo*gen	Inh-yvad
**CellROX deep red reagent**	Thermofisher	C10422
**Menadione**	Sigma	M5625-100G
**phorbol 12-myristate 13-acetate (PMA)**	Sigma	P1585
**C5b6**	CompTech	A122
**C7**	CompTech	A124
**C8**	CompTech	A125
**C9**	CompTech	A126
**Normal human serum (NHS)**	Generated in-house	N/A
**S1QE1. 1**	Sigma	SML1554
**S3QEL 2**	Sigma	ML1948
**Critical Commercial Assays**		
**JC-10 Mitochondrial Membrane Potential Assay Kit – Microplate**	Abcam	ab112134
**Amplex Red Hydrogen Peroxide/Peroxidase Assay Kit**	Invitrogen	A22188
**Cell-TiterGlo kit**	Promega	G7571
**Calcein AM cell viability kit**	Trevigen	4892-010-K
**L-Lactate Assay Kit (Colorimetric/Fluorometric)**	Abcam	Ab65330
**Human total IL-18 Duoset ELISA**	R&D Systems	DY318
**XF Cell Mito Stress test kit**	Agilent	103015-100
**XF Cell glycolytic rate test kit**	Agilent	103344-100
**Sequence-Based reagents**		
**IL-1β primers**	Applied Biosystems	Hs01555410m1
**PFKFB3 primers**	Applied Biosystems	Hs00998698_m1
**HIF1A primers**	Applied Biosystems	Hs00153153_m1
**PDK4 primers**	Qiagen	QT00003325
**TBP primers**	Applied Biosystems	Hs00427620_m1
**UBB primers**	Applied Biosystems	Hs00430290_m1
**B2M primers**	Applied Biosystems	Hs00187842m1
**Software and Algorithms**		
**GraphPad Prism**	GraphPad Software	http://www.graphpad.com/scientificsoftware/prism/
**Image J and Fiji**	ImageJ	https://imagej.net/Welcomand https://imagej.net/Fiji
**Zen Blue image analysis software**	Zeiss	https://www.zeiss.com/microscopy/int/products/microscope-software/zen.html
**Seahorse Wave Desktop Software**	Agilent	https://www.agilent.com/en/product/cell-analysis/real-time-cell-metabolic-analysis/xf-software/seahorse-wave-desktop-software-740897
**MetaboAnalyst**	MetaboAnalyst	https://www.metaboanalyst.ca/
**Perseus**	MaxQuant	https://maxquant.net/perseus/
**PANTHER classification system**	GeneOntology, Unifying Biology	http://pantherdb.org/
**Image StudioLite**	Licor	https://www.licor.com/bio/image-studio-lite/

## Materials and methods

### Primary monocyte differentiation and treatment

PBMCs were isolated from healthy human blood cones by gradient centrifugation. The PBMC layer was collected and monocytes isolated using CD14+ beads (Miltenyi Biotech) according to supplier’s protocol. For proteomics and metabolomics analysis, frozen human primary monocytes (Lonza) were used. Purified monocytes were plated and treated with GM-CSF (5 ng/mL) and cultured in RPMI-1640 (Life Technologies) with 5% fetal calf serum and 2mM L-glutamine for 6 days at 37°C, 5% CO2. All human biological samples were sourced ethically, and their research use was in accordance with the terms of the informed consents under an IRB/EC approved protocol.

On day 6, cells were washed once with RPMI media and sensitized to complement attack by addition of 7 µg/ml of anti-CD55, anti-CD59 and anti-HLA antibodies for 50 min at 37°C, 5% CO_2_. Antibody-sensitized cells were exposed to NHS or NHS that had been preincubated with an anti-C7 antibody for 30 min, on ice as a negative control for MAC formation (referred to as anti-C7 herein), or NHS alone at 37°C, 5% CO2 for the indicated amount of time. Subtlytic doses of MAC were characterised as <20% cell death (34, 35). Alternatively, MAC attack was induced using human purified proteins C5b6-9 for the extracellular H2O2 assay. Cells were incubated with anti-CD59 for 50 min at 37°C, 5% CO2. Antibody-sensitized cells were exposed to purified protein C5b6 for 10 min at room temperature, followed by addition of purified C7 for 15 min at 37°C, 5% CO2. C8 and C9 were then added sequentially and incubated at 37°C, 5% CO2 for the required time. C7, C8 and C9 were added in molar excess to the C5b6 concentration.

### Viability assays

Cell viability was measured by CellTitre-Glo (Promega) or calcein AM (Thermofisher). Both were used as per manufacturers protocols and data normalised to untreated control as 100% cell survival.

### MAC deposition assay

MAC deposition on cell lysates was measured by terminal complement complex (TCC) MSD developed in house. After MAC treatment, cells were washed twice with PBS and lysed with RIPA buffer for 20 min on ice. Briefly, the biotinylated C5b-9 (Abcam, biotinylated in-house) capture antibody was added to a MSD GOLD 96-Well Streptavidin Sector plate for 1 hr and washed three times with 0.05% Tween in PBS. All incubations were done at RT with shaking. Samples and standard curve (using human purified C5b6-9 for standards) were then added and left for 1 hr, followed by addition of ruthenylated anti-C6 (Quidel, ruthenylated in-house) detection antibody for 2 hrs. The plate was washed three times followed by addition of 2X Read buffer T (Mesoscale) and measured using MSD Sector 6000 Plate Reader.

### Lactate measurement

Macrophage supernatants were diluted accordingly and assayed for lactate measurement using the Fluorometric L-Lactate Assay Kit, according to the supplier’s protocol.

### Seahorse assays

The XF96 Seahorse analyzer was used to determine the bioenergetic profile of hMDMs. Briefly, 100,000 monocytes/well were differentiated in CellTak (22.3 μg/ml) coated 96XF seahorse plates. Prior to treatment, cells were washed twice with assay media XF RPMI medium, pH 7.4 supplemented with 2 mM L-glutamine, 25 mM glucose and 1 mM pyruvate. Cells were treated as required in assay media and during the 50 min incubation of antibody sensitization, plates were left in a CO2-free incubator. Cells were then exposed to NHS and either immediately inserted into the Seahorse analyzer or incubated for 4 h prior to assay. Cells were treated sequentially with 1 μM oligomycin, 1.2 μM carbonyl cyanide p-(trifluoromethoxy)phenylhydrazone (FCCP), and 1 μM antimycin A plus 1 μM rotenone for the Mitochondrial Stress Test and 1 μM antimycin A/1 μM rotenone and 50 mM 2-DG for the Glycolytic Rate Test. Data from all conditions were normalised using a post-run BCA assay. Six independent technical replicates per condition per donor were averaged. All parameters were calculated using Wave 2. 6. 1.

### Mitochondrial dynamics

hMDMs at 900000 cells/well in 27 mm Nunc glass bottom dishes (Thermofisher) were washed twice with imaging media (HBSS containing 5mM glucose, 20 mM HEPES and 1% BSA) and stained with 500 nM MitoTracker Red CMXRos for 15 min. Cells were washed twice and visualized on a Zeiss LSM880 confocal microscope before data capture. Cells were then treated with sublytic MAC, anti-C7 control or 5 μM ionomycin for 15 min in imaging media without BSA, and visualized on the above system with a Zeiss Plan-Apochromat 20x or 63x objective. For 20X, 9 images were taken for each condition and donor (3 x 3 tile), for 63X, 5 images per condition and donor were taken. Mitochondrial dynamics were quantified using the Yen threshold in the semi-automated analysis macro tool MiNA, used with Fiji/ImageJ software (46) (4 cells per condition per donor analyzed). The mitochondrial branch length mean values were exported, averaged and used as a measure for mitochondrial network morphology.

### Mitochondrial membrane potential assay

hMDMs at 100,000 cells/well in 96-well black microplates with clear bottom (Greiner) were washed in assay media (1X HBSS with 20 mM HEPES) and stimulated with sublytic MAC, anti-C7 control, as well as positive controls 5 μM ionomycin, 1. 2 μM FCCP or 1 μM rotenone for 30 min. Mitochondrial membrane potential was measured using the JC-10 Mitochondrial Membrane Potential Assay Kit – Microplate according to the supplier’s protocol. Fluorescence intensities were quantified using a plate reader at Ex/Em = 490/520 and 540/590 nm for ratio analysis. Ratios were normalised to untreated control as 100%. An increase in 520/590 nm ratio indicates a drop in mitochondrial membrane potential.

### Intracellular calcium assay

Intracellular Ca2+ of hMDMs was measured by monitoring of Fura-2 AM at 340/390_ex_/505_em_ nm. hMDMs at 100,000 cells/well in 96-well black microplates with clear bottom or at 900000 cells/well in 27 mm Nunc glass bottom dishes, for plate reader or confocal microscopy measurements, respectively, were pre-incubated with 3 μM Fura-2 reagent for 20 min in assay buffer (HBSS with 20 mM HEPES for plate reader, or HBSS with 5 mM glucose, 20 mM HEPES for imaging), washed twice in assay buffer and stimulated as required. Fluorescence quantification by plate reader measured the dual excitation ratio at 340/380 nm with emission at 505 nm. Ratios were normalised to untreated control as 100%. Alternatively, select conditions were visualized by a Zeiss LSM880 confocal system equipped with a Zeiss Plan-Apochromat 63x/1. 4 N. 63X images were taken for each condition. The excitation maxima of the dye shifts from 363 nm to 335 nm upon binding of the dye to Ca2+. Settings were selected to emit green fluorescence at 340 nm excitation, upon binding of Fura-2 to intracellular Ca2+.

### Western Blotting and peroxiredoxin assay

Cell lysates were generated in RIPA buffer containing protease inhibitors. Lysates were centrifuged (13,000 x g for 10 min at 4°C) and supernatants were diluted in NuPAGE® LDS Sample Buffer (4X), boiled at 90°C for 5 min and run in a 4-12% BisTris SDS-PAGE gel at 160V. Proteins were transferred onto nitrocellulose membranes using the iBlot2 device for 7 min before blocking with Odyssey® Blocking Buffer for 1 h at RT. Membranes were incubated overnight at 4°C with primary antibodies (rabbit anti-Prx3 (1:500), rabbit anti-gasdermin D (1:500) and mouse anti-β-actin (1:5000)). Membranes were washed 5 x 5 min in TBS-Tween (0. 1%) before incubation with secondary antibodies for 1 h at RT (IRDye® 800CW donkey anti-rabbit (1:7000) and IRDye® 680CW donkey anti-mouse (LI-COR) (1:10000)). Membranes were washed as above before visualization on Odyssey CLX. Western blot bands were quantified by measuring densitometry on Image J or ImageStudioLite. Bands for GSDMD were corrected against density of β-actin before comparison to UT or LPS-nigericin control. Prx dimerization was calculated by; % dimer = (SI dimer/((SI dimer + SI monomer)) x 100) % where SI= signal intensity Dimer was expressed relative to untreated control. Cell lysates for peroxiredoxin assay were generated by incubation of treated cells in 80 mM methylmethanethiosulfonate (MMTS) for 10 min at RT before HBSS wash and lysis in buffer (50 mM Tris, pH (8. 0), 150 mM NaCl 1% (v/v) Triton-X100, 0. 1% (w/v) SDS, 0. 5% (w/v) sodium deoxycholate), supplemented with 1:100 Protease Inhibitor Cocktail (Sigma), phenylmethanesulfonyl fluoride (0. 1 mM)) and MMTS (80 mM)).

### Real-time qPCR

Total RNA was extracted using QIAshredder Columns (Qiagen) and the RNeasy Mini kit (Qiagen). Purity and concentration of RNA was assessed using Nanodrop2000 UV-visible spectrophotometer. cDNA was generated using 30-200 ng/μl total RNA by a RT-PCR using the High-Capacity cDNA Reverse transcription kit (Applied Biosystems), according to the supplier’s protocol. quantitative PCR was run using SYBRGreen Mastermix (Applied Biosystems) on a QuantStudio 7 Flex System (Applied Biosystems). Primers specific for HIF-1α, IL-1β and PFKFB3 (Applied Biosystems) and PDK4 (Qiagen) were used. Gene expression was normalised to housekeeping genes UBB, B2M, and TBP (Applied Biosystems) using the Delta Ct method. Data were plotted as fold change obtained from relative quantification normalised to the untreated control.

### ROS production assays: Intracellular ROS and extracellular H2O2

For the CellROX assay, cells were treated as required including 20 uM menadione as a positive control for intracellular ROS. After treatment, cells were washed twice with HBSS and incubated with 5 μM CellROX™ Deep Red Reagent for 20 min at 37°C, 5% CO2. Plates were then washed twice with HBSS and fluorescence was measured in a plate reader. For the Amplex Red assay, which measured extracellular H2O2, cells were treated as required including 20 nM of PMA as a positive control using the Krebs–Ringer phosphate buffer (145 mM NaCl, 5. 7 mM sodium phosphate, 4. 86 mM KCl, 0. 54 mM CaCl2, 1. 22 mM MgSO4, 5. 5 mM glucose, pH 7. 35). Supernatants were collected and transferred in 96-well black plates with clear bottom. A H2O2 standard curve was prepared and all samples were mixed with 100 μM Amplex Red reagent and 0. 2 U/mL HRP working solution at 1:1 dilution. The plate was incubated for 30 min protected from light. Fluorescence was measured in a plate reader. For the CellROX assay, raw data obtained from fluorescence readings were normalised to untreated cells (0%) and to the positive control menadione (100%). For the Amplex red assay, raw data were extrapolated from the standard curve to H2O2 concentration in nM.

### Cytokine detection

Macrophage supernatants were assayed for the presence and quantification of IL-18 using commercial Duoset human ELISA kit (Applied Biosystems), according to the supplier’s protocol.

### ASC and MAC staining and confocal microscopy

80,000 monocytes/well were differentiated in Lab-tek slides (Life Technologies) as above. On d6, cells were treated with MAC or MAC + anti-C7 as above for 4 h before cells were washed twice in PBS, prior to fixation with 10% formalin solution (Sigma) for 15 min. Subsequently the cells were washed in PBS/0. 02% BSA/0. 02% NaN3 twice and were left in 200 ul of PBS/0. 02% BSA/0. 02% NaN3 for labelling with rabbit anti-ASC and anti-C9 (1/100 dilution) for 1h at RT. Cells were washed three times using PBS/0. 02% BSA/0. 02% NaN3 and labelled with the appropriate secondary antibody (1/500 dilution) for 1 h at RT. TOPRO-3 at 1 uL/mL was added for 5 min at RT prior to washing the cells three times using PBS/0. 02% BSA/0. 02% NaN3. Cells were imaged on a Carl Zeiss, Inc. LSM710 ELYRA P1 confocal microscope using a 1. 4 NA 63x Zeiss objective. The images were analyzed using Zen Blue image analysis software. The data presented are a representative image from at least 20 cells taken from three donors.

### Metabolomics analysis

hMDMs at 1 million cells/well in 24-well plates were sensitized to complement as usual and treated with a sublytic dose of NHS or left untreated for 4 h. Following treatment, supernatants were removed and the hMDMs rinsed with fresh assay media (RPMI with Glutamine) and frozen. For metabolite exaction, 75% 9:1 MeOH : CHCl3 was added directly to wells containing frozen cells. Cells from four technical replicates for each donor and sample type (NHS treated and untreated) were scraped from their respective wells, combined into Covaris adaptive focused acoustic tubes (for a total of 4 million cells per sample), and disrupted using a 2-minute lysis method on a Covaris S220 Focused Ulrasonicator (Peak Power - 200, Duty Factor - 10, Cycle/Burst - 200). Lysed samples were centrifuged at 5000 x g for 15 minutes at room temperature. Supernatant was split into two equal fractions and set on a Speedvac concentrator to dryness. Samples were reconstituted in either 3:1 MeCN:H2O or H2O+0. 1% formic acid for analysis by LC-MS/MS with hydrophilic interaction (HILIC) or reverse phase chromatography respectively.

All data was acquired with a Thermo-Fisher Scientific Ultimate 3000 Liquid Chromatograph coupled to a Q-Exactive Oritrap Mass Spectrometer with Heated Electrospray Ionization Source. For HILIC LC-MS/MS analysis, samples were analysed in positive and negative ion mode using a Phenomenex Luna NH2 analytical column (100 mm x 2 mm, 3 µm) held at room temperature with 10-minute linear gradient (A - 5% MeCN/20mM NH4CH2CO2/20mM NH4OH; B - MeCN) from 95 to 0% MeCN followed by 5 minute hold at a flow rate of 0. 400 mL/min. For reverse phase LC-MS/MS analysis, samples were analysed in positive ion mode only using a Waters Acquity BEH C18 analytical column (100 mm x 2. 1 mm, 1. 7 µm) held at 40°C with 4 minute linear gradient (A - H2O + 0. 1% formic acid; B - MeOH + 0. 1% formic acid) from 0. 5 to 70% MeOH, ramp to 98% MeOH 4. 5minutes, hold 98% to 5. 4 minutes at a flow rate of 0. 350 mL/min.

Mass spectrometric analysis was performed using data dependent acquisition. Full scan spectra were acquired at a scan range of 61 to 915 m/z at a resolution of 70,000 with an automatic gain control (AGC) of 1e6 ions and maximum injection time of 200 ms. Top 7 data dependent acquisition was employed with priority placed on a custom inclusion list built for known metabolite features. The custom inclusion list was derived from the analysis of neat standards part of the Mass Spectrometry Metabolite Library (IROA Technologies). Precursor ions were isolated with a quadrupole mass window of 1. 2 m/z and HCD fragmentation performed with stepped collision energy of 20, 30, and 45 V. MS/MS spectra were acquired at a resolution of 17,500 with an AGC target of 3e3 ions and a maximum injection time of 200 ms.

Raw data was aligned, integrated, and grouped using Thermo Compound Discoverer v3. 1. 0. 305. Deuterated L-Tryptophan, L-Phenylalanine, and Caffeine internal standards added to sample reconstitution solvents were used for data normalization. Peak annotation was based on the same database used to build the custom inclusion list for known metabolite features, and included retention time, m/z, and MS/MS data for > 500 primary metabolites. Peaks not annotated using the custom database were searched against m/z Cloud as an alternative approach to peak annotation. Statistical and pathway enrichment analysis, as well as data representation was performed using MetaboAnalyst 5. 0 (36, 37).

### Proteomics analysis

hMDMs were washed in assay media (RPMI with Glutamine) and treated with sublytic MAC, anti-C7 control or left untreated for 4 h. hMDMs were washed and lysed in the culture plates using a PreOmics kit and scraped into tubes. Protein quantitation was performed using a Pierce Rapid Gold BCA assay kit. (Thermo Scientific). Samples were reduced, alkylated, digested with trypsin/lys C and then labelled using 10-plex tandem mass tag (TMT) reagents (Thermo Fisher). Samples were combined to yield two TMT10 labelled sets, each containing one internal reference scaling (IRS) channel. The IRS sample was made by combining equal aliquots of all 18 samples and allowed for normalization across the TMT sets (75). Labelling efficiency and mixing ratios were tested by injecting a small amount of each TMT pool on a QExactive Orbitrap mass spectrometer (Thermo-Fisher Scientific). The two TMT-labelled pools were fractionated using hydrophilic interaction chromatography (HILIC) manual spin columns (Nest Group, Inc) into 4 fractions. The 4 fractions were dried under vacuum centrifugation and resuspended in 0. 1% (v/v) TFA in HPLC grade water.

Each fraction was separated by nanoflow HPLC (Easy nLC 1000, Thermo-Fisher Scientific) using a C18 PepMap trap column (2cm x 175µm ID, PepMap C18, 3µm particles, 100 Å pore size) and a 25 cm EasySpray column (PepMap 25cm x 75µm ID, C18, 2 µm particle size, 100 Å pore size) with a linear gradient (2-28% MeCN, 0. 1% FA) over 240 minutes at 300 nL/min. Mass spectrometric analysis was performed on a QExactive Orbitrap mass spectrometer (Thermo-Fisher Scientific) operated in positive ionization mode with data dependent acquisition. Full scan spectra were acquired at a scan range of 400 to 2000 m/z at a resolution of 70,000, with an automatic gain control (AGC) of 1e6 ions and a maximum injection time of 200 ms. The 10 most intense precursor ions were isolated with a quadrupole mass filter of 2. 0 m/z and collision induced dissociation (CID) fragmentation was performed with a stepped collision energy of 24, 27, 30 V. MS/MS spectra were acquired at a resolution of 35,000 with an AGC target of 5e4 ions and a maximum injection time of 200 ms.

Protein identification for the total TMT labelled data set was performed using MaxQuant 1. 6. 10. 0. The reporter ion MS2 type set as TMT10plex; trypsin/P set as the enzyme; fixed modification PreOmics iST-NHS (C); variable modification Oxidation (M); maximum 2 missed cleavages. Searches were conducted using the human Uniprot database. Quantification of proteins uses razor peptides with a minimum count of 2. Contaminants and reverse hits were removed from the data sets prior to analyses. The data were normalised within and across the TMT sets prior to statistical analyses (75). A total of 2885 proteins were identified of which 1982 were quantified. Statistical analyses were performed using Perseus (version 1. 6. 15. 0) (76). Perseus was used for generation of hierarchical clustering of normalized protein intensities (z-score) for significantly regulated proteins (ANOVA permutation=based FDR <0. 05) and for the one-way ANOVA, FDR corrected, with post-hoc Tukey’s test (p<0. 05 significant for both MAC vs untreated and MAC vs AC7) significant proteins from which a list of MAC regulated proteins was generated and used for supplementary tables, panel of proteins of interest (mainly mitochondrial proteins) and GO statistical enrichment testing for GO biological processes provided by PANTHER classification system online software (using ID lists with associated expression value z-scores).

### Quantification and statistical analysis

#### Statistical analysis

Data represent the mean ± SEM. Differences between groups are analysed using an unpaired student’s t-test with Welch’s correction for unequal SDs or 1-way ANOVA with post-hoc Tukey’s test as required, using GraphPad Prism 7 software, unless indicated otherwise. *p < 0.05, **p < 0.01, ***p < 0.001, ****p < 0.0001. For all experiments n = number of separate donors as highlighted in the legend.

## Data availability statement

The datasets presented in this study can be found in online repositories. The names of the repository/repositories and accession number(s) can be found below: ProteomeXchange Consortium *via* PRIDE (77) dataset identifier PXD027316. The metabolomics dataset is available on Metabolomics Workbench under the Project ID PR001213.

## Author contributions

LMB and KT carried out study conception and design. GJ-D designed, performed and analyzed most experimental work. LMB assisted with experimental work and data analysis. JK and CK performed metabolomics analysis. RP-H, ED, and RA were involved in proteomics analysis NG aided with statistical analysis (except for metabolomics and proteomics analysis). LMB and KT supervised project. MT and E-MN assisted with project supervision. LMB contributed to funding acquisition/project administration. All authors contributed to the article and approved the submitted version.

## Acknowledgments

We acknowledge the membership of the Immunology Research Unit (GSK) and Immunology Network group (GSK) including G. Tannahill, and E. Koppe for project discussions and operational support, as well as Cardiff University.

## Conflict of interest

Authors JK, RP-H, RA, ED, E-MN, CK, FZ, NG, LM, and LMB are employees and shareholders of GSK. All authors, except GJ-D, KT and MT, were employed by GSK at the time of the work. The Immunology Network at GSK funded GJ-Ds PhD studentship and research costs. The Immunology Network at GSK also funded secondments for KT and MT to undertake this research at GSK.

The authors declare that the study was funded by GSK. The funder was involved in the study design, collection, analysis, interpretation of data, the writing of this article or the decision to submit it for publication.

## Publisher’s note

All claims expressed in this article are solely those of the authors and do not necessarily represent those of their affiliated organizations, or those of the publisher, the editors and the reviewers. Any product that may be evaluated in this article, or claim that may be made by its manufacturer, is not guaranteed or endorsed by the publisher.

## Supplementary material

Supplementary Figure 1
**(A)** CellTiter-Glo and Calcein AM assays to determine hMDMs viability upon antibodies (anti-CD55, CD59, HLA) sensitisation and stimulation with increasing concentrations of NHS for 1 hour. n=3. **(B)** CellTiter-Glo assay to determine hMDMs viability upon antibodies (anti-CD55, CD59, HLA) sensitisation and stimulation with increasing concentrations of NHS for 1, 4 and 24 hours. **(C)** Sublytic levels of NHS (6% NHS) at 1 hour (corresponding to 80% survival) 4 and 24 hours (corresponding to 70-80% survival) (n=3). Error bars represent mean +/- S. E. M.

Supplementary Figure 2
**(A)** Cell viability measurement by CellTiter-Glo or Calcein AM from hMDMs treated with antibodies (anti-CD55, CD59, HLA) and increasing concentrations of NHS for 1 hour, to stablish sublytic complement NHS concentration (80% survival) for each donor (n=6 independent donors). **(B)** Heat map resulting from targeted metabolomics analysis comparing NHS (named as MAC here) between Untreated conditions for each donor (e. g. Untreated 1-6 referring to each donor labelled). **(C)** Enrichment pathway analysis using p value and enrichment ratio to rate the most upregulated (right) and downregulated (left) metabolic pathways vs Untreated control. **(D)** Raw values of peak area from selected metabolites (n=6). Statistical significance in **(D)** was assessed by unpaired student’s t-test with Welch’s correction for unequal SDs. Error bars represent +/- S. E. M

Supplementary Figure 3 Proteomics analysis downstream of sublytic complement attack on hMDMs. **(A)** Hierarchical clustering of normalized protein intensities (z-score) for significantly regulated proteins (ANOVA permutation=based FDR < 0.05) (N= 724 regulated proteins). Mitochondrial proteins of interest are highlighted in their corresponding cluster. **(B)** Expression profiles of proteins in three selected clusters corresponding to **Supplementary Figure 5A** showing distinct behaviours: 1) downregulation in MAC stimulated and anti-C7 stimulated samples; 2) downregulation in MAC treated samples and rescue to UT baseline with anti-C7; and 3) downregulation in MAC treated samples and recovery with anti-C7. **(C)** Top 10 list of MAC downregulated pathways from proteomics data using statistical enrichment test for GO biological processes analysis (N= 363 downregulated proteins). See methods section. **(D)** hMDMS treated with MAC (+/- anti-C7) or LPS for 24 hours. Gene expression of HIF-1a, IL-1B, PFKFB3 and PDK4 (iPDH; inhibitor of PDH) normalised to β-actin, HPRT and TBP and expressed as fold change relative to unstimulated hMDMs (n=5).

Supplementary Figure 4
**(A)** hMDMs were differentiated in Cell-Tak coated 96XF seahorse plates at 6 different cell densities. Oxygen consumption rate (OCR) was measured under the Mitochondrial Stress Test in XF96 Seahorse. Cell survival was also measured using Celltiter-Glo assay upon increasing doses of NHS to determine sublytic MAC concentration of NHS at 80% survival in seahorse plates (n=3). **(B)** Parameters from the seahorse Glycolytic rate test shown in Fig. 3A and the Mito stress test shown in Fig. 3B (n=4). **(C–E)** hMDMs stimulated with MAC, anti-C7 + MAC or 5 mM Glycine pre-incubated for 50 min plus MAC for 1 hour. **(F, G)** hMDMs stimulated with MAC, anti-C7 + MAC or 5 mM Glycine pre-incubated for 50 min plus MAC for 24 hour before Mitochondrial Stress Test by Seahorse XF. 0. 5 µM rotenone/antimycin A was added prior to start of test in R/AA condition. Parameters are presented in G. Seahorse measurements: GlycoPER from glycolytic rate test **(C)** (n=3) and mito stress test **(D, F)** (n=4), respectively, were measured, and parameters presented in **(E, G)**. Statistical analysis in **(B, D, G)** was assessed by 1-way ANOVA with post-hoc Tukey’s test. Error bars represent ± S. E. M.

Supplementary Figure 5
**(A)** WB quantification from , Prx3 dimerization measured by Image J and normalised to untreated (n=3). **(B, C)** Cells sensitised with neutralising anti-CD59 antibody and stimulated with purified components of MAC. **(B)** 6 µg/ml of C5b6 plus one molar excess of C7, C8 and C9, viability was measured by CellTiter-Glo (n=4). **(C)** MAC deposition after set up as in **(B)** measured by TCC MSD in cell lysates (n=3). **(D)** IL-18 release by hMDMs detected by IL-18 ELISA of supernatants of hMDMs in 1 million cells per condition (24-well plates) (same 3 donors from which contains additional positive and negative controls) stimulated with MAC for 3 hours with inhibitors 2-DG (5mM), HA (10 μM), and rotenone (0. 5 μM). Statistical analysis performed on **(B–D)** was assessed by unpaired student’s t-test with Welch’s correction for unequal SDs. Error bars represent ± S. E. M.
