# Supplementary material for: Complement membrane attack complex is an immunometabolic regulator of NLRP3 activation and IL-18 secretion in human macrophages
Source: Front Immunol. 2022 Sep 27;13:918551. doi: 10.3389/fimmu.2022.918551 (PMC9554752; doi:10.3389/fimmu.2022.918551)
Supplement: Supplementary file 1 [file Table_1.pdf]

Supplementary table 1: Proteins upregulated by MAC stimulation. Data obtained from proteomics analysis, numbers represent normalized protein intensities (z-score) for significantly MAC-regulated proteins (one-way ANOVA, FDR corrected, with post-hoc Tukey's test p<0.05 significant for MAC vs Untreated and MAC vs anti-C7 + MAC. N= 99 proteins).

**Protein ID (Fasta headers)**	**Untreated**	**MAC**	**aC7 + MAC**
SERPING1	-0.71255	1.338117	-0.62557
UBR2	-0.73284	1.33567	-0.60283
A1BG	-0.65136	1.309381	-0.65802
HP	-0.85078	1.306739	-0.45596
HPX	-0.81053	1.305941	-0.49541
C5	-0.73911	1.301053	-0.56194
ITIH2	-0.71568	1.300694	-0.58501
SERPINA3	-0.74811	1.29881	-0.5507
APOA1	-0.86949	1.296986	-0.4275
SERPIND1	-0.81375	1.296947	-0.4832
APOB	-0.76986	1.296218	-0.52636
CLU	-0.87726	1.291073	-0.41381
CFB	-0.77141	1.290904	-0.51949
AHSG	-0.8075	1.284994	-0.47749
SERPINA1	-0.80279	1.279609	-0.47682
PPBP	-0.55956	1.276812	-0.71725
PLG	-0.79538	1.272229	-0.47685
RBM25	-0.52358	1.271531	-0.74795
ITIH1	-0.72299	1.262638	-0.53965
PON1	-0.77602	1.259534	-0.48351
CFH	-0.77569	1.249677	-0.47399
SPNS1	-0.69375	1.24736	-0.55361
HBB	-0.71069	1.240967	-0.53028
IGHG4	-0.87111	1.240902	-0.36979
CP	-0.75698	1.238097	-0.48112
A2M	-0.77889	1.228924	-0.45003
ITIH4	-0.85255	1.218072	-0.36552
C1S	-0.93564	1.214084	-0.27844
C3	-0.79324	1.206814	-0.41358
C4B	-0.95619	1.206672	-0.25048
KNG1	-0.73922	1.204148	-0.46493
GC	-0.65353	1.202312	-0.54878
IGHV3-7	-0.64889	1.200161	-0.55127
IGHM	-0.91065	1.194987	-0.28434
ECHS1	-0.74196	1.190987	-0.44903
TNFAIP8	-0.40302	1.176817	-0.7738
ARL3	-0.54583	1.174091	-0.62826

TPM3	-0.61508	1.173412	-0.55834
F2	-0.97608	1.168344	-0.19227
APOA2	-1.02985	1.159158	-0.1293
IGHG2	-0.87977	1.146207	-0.26644
SERPINC1	-0.89485	1.137588	-0.24274
C4A	-0.98477	1.135462	-0.15069
APOC1	-0.97363	1.134989	-0.16136
PAFAH1B2	-0.63681	1.131831	-0.49502
APOC3	-0.8344	1.126326	-0.29193
HBA1	-0.63751	1.114931	-0.47742
IGKC	-0.89884	1.112496	-0.21366
PTPN11	-0.36103	1.109567	-0.74854
APOA4	-1.04336	1.109534	-0.06618
PRKAR1A	-0.40122	1.10773	-0.70651
NDUFB4	-0.69622	1.10625	-0.41003
IGHA1	-0.83461	1.105246	-0.27064
TXNDC12	-0.31087	1.103287	-0.79242
VTN	-0.73897	1.102314	-0.36334
TTR	-0.77598	1.089919	-0.31394
GLUD1	-0.32479	1.089896	-0.7651
IGHG3	-0.95666	1.087918	-0.13126
HRG	-0.76665	1.078019	-0.31137
PSMD11	-0.45692	1.066679	-0.60976
TRIM28	-0.57373	1.06625	-0.49252
OS9	-0.65108	1.064728	-0.41365
SMU1	-0.38585	1.063113	-0.67727
HMGB2	-0.67643	1.060792	-0.38437
GORASP2	-0.30949	1.057231	-0.74774
MTHFD2	-0.22656	1.050459	-0.8239
VPS25	-0.43193	1.044245	-0.61232
DPM1	-0.36463	1.042769	-0.67814
ACTR2	-0.893	1.033825	-0.14083
IGLL5	-0.82442	1.032247	-0.20782
MACF1	-0.42152	1.029411	-0.60789
IGHG1	-0.81882	1.028326	-0.20951
SMC1A	-0.89331	1.01618	-0.12287
STIM1	-0.15589	1.011497	-0.85561
CHMP5	-0.61915	1.010454	-0.3913
RRAGC	-0.49623	1.005379	-0.50915
LAMTOR2	-0.83316	0.994936	-0.16178
TMED10	-0.20135	0.993748	-0.7924
LRCH1	-0.10352	0.987973	-0.88446
PSMD3	-0.39866	0.977633	-0.57898
RPS6KA3	-0.21328	0.973116	-0.75984
DUSP3	-0.31411	0.973027	-0.65892
IGLC3	-0.79193	0.967287	-0.17536

RPA1	-0.32039	0.95364	-0.63325
CD97	-0.39168	0.951541	-0.55986
AKR1C3	-0.35753	0.950579	-0.59305
AUP1	-0.3991	0.947708	-0.54861
EIF3I	-0.35877	0.944758	-0.58599
BAG6	-0.75017	0.944371	-0.1942
DUSP23	-0.7163	0.94184	-0.22554
RARS	-0.5214	0.932948	-0.41155
C1QB	-0.54084	0.924238	-0.3834
SRSF11	-0.44116	0.921527	-0.48036
VPS35L	-0.55743	0.917266	-0.35983
MDH1	-0.40756	0.917197	-0.50964
SRI	-0.46669	0.916254	-0.44956
PSMC6	-0.37077	0.91443	-0.54366
MYDGF	-0.51512	0.914419	-0.3993
CAMSAP1	-0.49145	0.867114	-0.37566

Supplementary table 2: Proteins downregulated by MAC stimulation. Data obtained from proteomics analysis, numbers represent normalized protein intensities (z-score) for significantly MAC-regulated proteins (one-way ANOVA, FDR corrected, with post-hoc Tukey's test p<0.05 significant for MAC vs Untreated and MAC vs anti-C7 + MAC. N= 363 proteins).

Protein (Fasta headers)	Untreated	MAC	aC7 + MAC
CLPTM1	0.41019	-0.85836	0.448172
DHX9	0.421147	-0.86868	0.447537
MAP2K1	0.485653	-0.87155	0.385902
SWAP70	0.384621	-0.87212	0.487497
NT5C2	0.413777	-0.88353	0.469755
ARPC5	0.458051	-0.88551	0.427464
LDHA	0.35808	-0.88683	0.528751
BASP1	0.365377	-0.89077	0.525391
ARRB2	0.559942	-0.89218	0.332236
TGM2	0.453488	-0.89229	0.438807
COX6B1	0.485838	-0.89302	0.407183
CORO1B	0.327894	-0.89303	0.565137
MIEN1	0.356129	-0.89409	0.537965
ACAD9	0.510328	-0.89433	0.384005
GK3P	0.367132	-0.8953	0.528171
UBE2V1	0.348103	-0.89563	0.547527
COPB1	0.598134	-0.89838	0.300245
DNM2	0.513394	-0.90021	0.386811
PSMD8	0.436484	-0.901	0.464518
HSP90AA1	0.322753	-0.90248	0.579724

CD63	0.307394	-0.9049	0.597503
NONO	0.469664	-0.90536	0.435694
SOD2	0.337906	-0.90757	0.569666
ATIC	0.475772	-0.90771	0.431935
CTSA	0.478559	-0.90854	0.429978
COX7C	0.4097	-0.90864	0.498937
ATP6V0D1	0.371056	-0.9099	0.53884
ATP2A2	0.546152	-0.91376	0.367609
NIBAN1	0.611739	-0.91639	0.304653
EEF1E1	0.319473	-0.91656	0.597085
UBE2K	0.565429	-0.91762	0.352195
GYG1	0.455294	-0.91906	0.463768
SEPTIN11	0.289195	-0.9196	0.630409
ACAA1	0.379541	-0.92017	0.540625
SLC25A13	0.444474	-0.92045	0.475973
NAPA	0.403849	-0.92064	0.516795
GRB2	0.244387	-0.92108	0.67669
STOM	0.521715	-0.92443	0.402719
HMGCL	0.275706	-0.92826	0.652554
LMNB1	0.486815	-0.93022	0.443401
DDOST	0.383457	-0.93025	0.546798
IL16	0.414642	-0.93142	0.516773
IDH3A	0.358325	-0.93144	0.573115
SDC2	0.480108	-0.93247	0.452359
FLII	0.556064	-0.93283	0.376769
GFPT1	0.699298	-0.93328	0.233985
COMMD9	0.552278	-0.93352	0.38124
SEC31A	0.371101	-0.93359	0.562491
RDX	0.341887	-0.93418	0.592291
EHD4	0.534113	-0.93682	0.402709
COPS5	0.414584	-0.93698	0.522394
TMA7	0.457998	-0.93849	0.48049
PSMD12	0.191728	-0.93965	0.747926
YWHAQ	0.232763	-0.94356	0.710802
SH3GLB1	0.787504	-0.94374	0.15624
ATP5IF1	0.414002	-0.94589	0.531886
NMT1	0.420713	-0.94804	0.52733
CHMP1A	0.424986	-0.9528	0.527815
SCARB2	0.53267	-0.95286	0.420193
SNRPD2	0.039838	-0.95485	0.915017
RPLP2	0.245535	-0.9554	0.709861
MTPN	0.311485	-0.95563	0.644149
MAGOH	0.391775	-0.95583	0.564059
GOLGB1	0.279951	-0.95675	0.676797
CD14	0.015438	-0.95703	0.941591
PSMB10	0.39909	-0.95896	0.559873

SEPTIN7	0.370236	-0.96029	0.590049
DERA	0.234476	-0.96046	0.725986
CAPRIN1	0.873393	-0.9607	0.087305
GPX1	0.425636	-0.96119	0.535553
S100A6	0.12674	-0.96232	0.835581
PDCD6IP	0.383048	-0.96406	0.581011
RNPEP	0.496114	-0.9652	0.469082
YWHAE	0.148421	-0.96561	0.817194
NUCB2	0.143036	-0.96679	0.823754
PTGR1	0.430937	-0.96749	0.536555
RBBP7	0.543828	-0.9688	0.424967
MAPRE2	0.708279	-0.9696	0.261324
PDCD5	0.187875	-0.97066	0.782785
MOSPD2	0.419879	-0.97068	0.550804
CALM3	-0.04227	-0.97089	1.013161
EPRS	0.53987	-0.97175	0.431876
SYNCRIP	0.324429	-0.97198	0.647551
ECPAS	0.631895	-0.97312	0.34122
AHNAK	0.108581	-0.97356	0.864976
RAP1B	0.797079	-0.97396	0.176878
CARHSP1	0.536537	-0.97511	0.438569
ARL8B	0.420487	-0.97528	0.554794
BSG	0.299224	-0.9761	0.676875
CAB39	0.238143	-0.97774	0.739594
PDXK	0.48233	-0.97833	0.496004
MAT2A	0.495935	-0.98071	0.48478
IPO5	0.674683	-0.98127	0.30659
FCER1G	0.117962	-0.98215	0.86419
RPLP0	0.812724	-0.98294	0.170215
HACD3	0.882775	-0.98341	0.100638
NPC2	0.212605	-0.98352	0.770918
PECAM1	0.639515	-0.98357	0.344058
LYPLA1	0.623819	-0.9836	0.359782
TNPO3	0.491523	-0.98404	0.49252
RBM47	0.282262	-0.98413	0.701873
ASAH1	0.217472	-0.98445	0.766977
COPZ1	0.142011	-0.98476	0.84275
TMED9	0.452915	-0.98568	0.532763
TPI1	-0.011	-0.9864	0.997397
HNRNPDL	0.185537	-0.98676	0.801224
RPS7	0.443002	-0.98756	0.544554
DYNLL1	0.687576	-0.98772	0.300148
PTPRE	0.578776	-0.9883	0.409527
HYOU1	0.667319	-0.98953	0.322209
GLG1	0.746022	-0.98964	0.243618
IARS2	0.22923	-0.98974	0.760506

LRRFIP1	0.203442	-0.99111	0.787672
WASHC5	0.692021	-0.99373	0.301707
ST13	0.119445	-0.99472	0.87527
PRKCD	0.706945	-0.99708	0.290131
ACOX1	0.703793	-0.99716	0.293365
PPP1CB	0.924413	-0.9975	0.073091
SNRPF	0.02904	-0.99754	0.968504
SERBP1	0.192554	-0.99813	0.80558
PABPC4	0.280198	-0.99935	0.719155
LIMS1	0.001825	-0.9994	0.997571
CSRP1	0.401714	-1.00044	0.598728
RHOA	-0.16262	-1.00098	1.163601
EIF3G	0.381594	-1.00223	0.620634
RPL8	0.707964	-1.00272	0.294758
SLC8A1	0.364758	-1.00511	0.640349
MRPL12	0.279662	-1.00608	0.726421
UBE2N	0.613655	-1.00659	0.392934
SARS	0.520637	-1.00745	0.486816
PSMA7	0.238006	-1.00766	0.769657
DNAJC7	0.699951	-1.00817	0.308216
AASDHPPT	0.31844	-1.00842	0.689981
ATG7	0.427036	-1.00864	0.581606
GABARAPL2	0.30433	-1.00878	0.704447
CD84	0.691026	-1.00881	0.31778
EEF1D	0.277812	-1.01092	0.73311
VCP	0.510761	-1.01199	0.501225
PLCB2	0.504676	-1.01392	0.509249
XRCC6	0.443992	-1.01405	0.570053
XPO7	0.884494	-1.01432	0.129823
MAT2B	0.577122	-1.01443	0.437312
PSMA6	0.36092	-1.01699	0.656074
HNRNPD	0.046359	-1.01869	0.972333
XRCC5	0.358418	-1.01882	0.660399
RAD23B	0.328643	-1.02002	0.691374
CANX	0.1716	-1.02124	0.849642
PSMC3	0.642891	-1.0216	0.37871
HERC4	0.912357	-1.02214	0.109779
PSMC4	0.207166	-1.0223	0.815135
ASNA1	0.747502	-1.02278	0.275278
EIF3M	0.006781	-1.02499	1.018207
ARHGAP45	0.771879	-1.02523	0.253351
ARHGAP30	0.778453	-1.02617	0.24772
HYPK	0.224363	-1.02617	0.801811
EIF3H	0.645565	-1.02669	0.381127
EEF1B2	0.190321	-1.02728	0.836963
VAV1	1.028551	-1.02901	0.000462

PSAP	0.248172	-1.03205	0.783874
PRKACA	0.600218	-1.03279	0.432568
CALR	0.294456	-1.03381	0.739358
CDC37	0.07877	-1.03439	0.955624
SAR1A	0.256372	-1.03626	0.77989
CNPY3	0.617823	-1.03639	0.418569
RAB14	0.499145	-1.03681	0.537661
C5AR1	0.371305	-1.03769	0.666386
HNRNPF	0.716899	-1.0391	0.322198
ENSA	0.409574	-1.0404	0.630823
HNRNPUL1	0.443054	-1.04041	0.597353
DOCK8	0.630626	-1.04062	0.409997
CLIC4	0.266166	-1.0413	0.775135
TMEM43	0.602002	-1.04158	0.439574
NDRG1	0.530408	-1.04238	0.511973
CSTB	-0.00483	-1.04252	1.04735
CLTB	0.304718	-1.04368	0.738959
PRPS2	0.540434	-1.04416	0.50373
SUMO3	0.334488	-1.04463	0.710146
ILF2	0.849995	-1.0458	0.19581
RPS15	0.402999	-1.04873	0.645732
MYL6	0.561909	-1.04879	0.48688
RANBP1	0.224651	-1.04897	0.82432
UCHL3	0.27092	-1.05073	0.779813
CLTCL1	0.389523	-1.051	0.661479
TPMT	0.415074	-1.05728	0.642209
CAST	0.211479	-1.05742	0.845938
SUMF2	0.280127	-1.05775	0.77762
PPA1	0.540331	-1.05862	0.518288
EIF3F	0.231252	-1.06087	0.829622
SF3B1	0.621322	-1.06124	0.439922
SNX6	0.648097	-1.06171	0.413616
RPL30	0.817284	-1.0618	0.244514
DARS	0.628788	-1.06218	0.43339
KIF5B	0.678668	-1.06348	0.384814
TXNL1	0.214923	-1.06424	0.849313
PSMB1	0.556783	-1.06432	0.507538
LSP1	0.172491	-1.06514	0.892646
BROX	0.470831	-1.06593	0.595099
UFM1	0.30246	-1.06631	0.763854
GDI2	0.537056	-1.06663	0.529574
SRP9	0.324722	-1.06747	0.74275
PSMA4	0.089094	-1.06901	0.979917
SET	0.164126	-1.06929	0.905165
CSTA	0.347676	-1.06934	0.721663
ALDH3A2	0.492441	-1.06943	0.57699

ARMT1	0.271806	-1.06948	0.797678
SIRPA	0.435267	-1.06986	0.634593
CBR1	0.314085	-1.0706	0.756516
SNAP23	0.320483	-1.07191	0.751426
ANPEP	0.302605	-1.07196	0.769354
STRAP	0.470488	-1.07282	0.602332
MANF	0.403991	-1.07321	0.669219
SUB1	0.12232	-1.07404	0.951719
TRIM25	0.707188	-1.0748	0.367611
STIP1	0.29284	-1.07547	0.782625
TBCB	0.564753	-1.07553	0.510777
SPCS2	0.551356	-1.07642	0.525065
PTPA	0.537846	-1.07875	0.540902
EIF3D	0.60736	-1.07889	0.47153
ARF5	0.57247	-1.08131	0.508844
GGCT	0.462264	-1.08255	0.620288
EIF3B	0.551611	-1.08256	0.530946
PSME3	0.342028	-1.08421	0.74218
ALDH3B1	0.357392	-1.08479	0.727397
PCBP1	0.615402	-1.08554	0.470136
PPP1CA	0.165659	-1.0867	0.921044
CLIC1	0.141116	-1.08751	0.946389
UNC45A	0.708702	-1.08842	0.379717
COMMD7	0.678376	-1.08983	0.411449
DNAJA2	0.596779	-1.09069	0.493913
KCTD12	0.649281	-1.09437	0.445093
ADSL	0.369836	-1.09448	0.724644
YARS	0.327476	-1.09605	0.768571
AK3	0.540709	-1.09623	0.555524
SLC1A4	0.473417	-1.09637	0.622954
OLA1	0.606372	-1.0969	0.490525
COPB2	0.49227	-1.09705	0.604784
ATP1B3	0.455278	-1.09952	0.644245
AP2B1	0.73121	-1.09972	0.368511
CD81	0.374403	-1.10004	0.725635
HSPA4	0.370847	-1.10051	0.729667
JPT1	0.466261	-1.10095	0.634688
EEF1G	0.74887	-1.10168	0.352814
PSMA1	0.289202	-1.10323	0.814031
SLC30A1	0.553537	-1.10361	0.550075
ATOX1	0.240053	-1.10377	0.863714
IQGAP1	0.855377	-1.10443	0.249056
CAP1	0.45661	-1.10567	0.649062
TKT	0.301797	-1.10575	0.803954
PSMA5	-0.06015	-1.10593	1.166083
CRIP1	0.413432	-1.10658	0.693147

RAB10	0.372529	-1.10684	0.734315
HNRNPAB	0.298485	-1.11006	0.811577
ITGB1	0.173361	-1.11016	0.936799
NAA15	0.611674	-1.11081	0.499131
CSK	0.71961	-1.11224	0.392634
LASP1	0.544955	-1.11446	0.569503
ANXA11	0.525823	-1.11582	0.589998
CDS2	0.532589	-1.11599	0.583399
RTN4	0.765684	-1.11719	0.35151
BTF3	0.33832	-1.11728	0.77896
TOR1AIP1	0.327103	-1.11795	0.790845
CAPNS1	0.069497	-1.11888	1.049379
PDAP1	0.360801	-1.11956	0.75876
NDUFA5	0.611642	-1.11962	0.507982
EPB41L3	0.562618	-1.12049	0.557875
GRPEL1	0.284751	-1.12421	0.839454
G3BP1	0.555027	-1.12444	0.56941
CAPZA1	0.36142	-1.12568	0.764257
ATP6V1H	0.502977	-1.12681	0.623832
HNRNPK	0.582447	-1.12891	0.54646
CDV3	0.245218	-1.13024	0.885024
ARF1	0.447917	-1.13124	0.683327
HSPB11	0.558653	-1.13198	0.573322
LCP1	0.429345	-1.13277	0.703422
DNAJB1	0.639762	-1.13447	0.494707
PHB	0.743639	-1.13479	0.391147
S100A11	0.213611	-1.13519	0.921577
CRABP2	0.540283	-1.13625	0.595971
AHCY	0.685087	-1.13758	0.452493
CHMP4B	0.600087	-1.13782	0.537736
TFG	0.311523	-1.13838	0.826854
PPP1R12A	0.149796	-1.14025	0.99045
SGTB	0.347565	-1.14056	0.79299
PDHB	0.52555	-1.14177	0.616219
TFRC	0.953561	-1.14227	0.188704
SLC39A11	0.497139	-1.14246	0.645317
ATP6V1E1	0.614883	-1.14269	0.527804
TBCA	0.313788	-1.14465	0.830863
CCT6A	0.667994	-1.14551	0.477518
EFHD2	0.511722	-1.14552	0.633795
ALCAM	0.49633	-1.14782	0.65149
GBP2	0.470996	-1.14916	0.678162
KLC1	0.560986	-1.14984	0.58885
LPP	0.399825	-1.15076	0.750937
CAND1	0.702859	-1.15122	0.448365
BRK1	0.423172	-1.15499	0.731821

MARCKS	0.40606	-1.15657	0.750508
CMPK1	0.312246	-1.15695	0.844706
WDR11	0.573054	-1.15863	0.585574
EIF1AX	0.463102	-1.15911	0.696011
TMED2	0.569581	-1.1595	0.589921
KHSRP	0.580556	-1.1604	0.579842
SNX5	0.50865	-1.16063	0.651977
PURB	0.496986	-1.16358	0.666593
CBX3	0.449747	-1.1644	0.714658
PTRHD1	0.501413	-1.16441	0.663001
ELOB	0.482243	-1.16468	0.682436
BTF3L4	0.326005	-1.16488	0.838877
RP2	0.443303	-1.16786	0.72456
STAT6	0.812484	-1.16794	0.35546
TMED5	0.481758	-1.16962	0.687862
AARS	0.434878	-1.17183	0.736948
HSPA1B	0.702669	-1.17451	0.47184
FUS	0.488013	-1.17544	0.687423
CCT5	0.595062	-1.17653	0.581472
SLC9A3R1	0.565906	-1.17723	0.611328
UFC1	0.458717	-1.17962	0.720904
NME2	0.382623	-1.18109	0.798468
ISOC1	0.398895	-1.18441	0.785514
CHD4	0.468883	-1.18471	0.715825
SAR1B	0.451921	-1.18513	0.733205
CSE1L	0.635746	-1.18621	0.550463
PSMD14	0.515069	-1.18913	0.674064
ARL8A	0.406843	-1.19083	0.783987
SEC13	0.58594	-1.19172	0.605783
NSFL1C	0.357201	-1.19711	0.839905
DEK	0.601948	-1.19838	0.596432
SCFD1	0.427559	-1.19959	0.772026
ADH5	0.406524	-1.20248	0.795955
WASF2	0.458507	-1.20355	0.745044
CYFIP1	0.61934	-1.2037	0.58436
UBA52	0.481535	-1.20476	0.723228
PPP2R1A	0.678503	-1.20911	0.53061
SSB	0.439882	-1.21053	0.770648
ZYX	0.366826	-1.21338	0.846556
CACYBP	0.639687	-1.21834	0.578657
PITPNB	0.660359	-1.21846	0.558104
REEP5	0.387474	-1.21912	0.831647
GLO1	0.462582	-1.22192	0.759339
SPAG9	0.452051	-1.22338	0.771326
PI4K2A	0.654234	-1.22505	0.570819
CAPN1	0.675083	-1.22717	0.552083

PLAA	0.567759	-1.22841	0.660651
EIF3E	0.37	-1.22861	0.858612
NSUN2	0.510029	-1.22946	0.719433
CD82	0.534568	-1.23374	0.699176
PTPN6	0.595972	-1.23718	0.641207
PPP3R1	0.627218	-1.23799	0.610777
HMGN1	0.650966	-1.24114	0.590176
UBLCP1	0.535318	-1.24301	0.707688
ENOPH1	0.613466	-1.24411	0.63064
EIF2S2	0.417241	-1.24624	0.828997
PAK2	0.6063	-1.2467	0.640403
GMPS	0.738126	-1.24805	0.509928
SEC62	0.491545	-1.25127	0.759724
CCT3	0.743567	-1.26067	0.517099
STT3B	0.731197	-1.26484	0.533641
RAB35	0.432714	-1.26575	0.833037
GPI	0.480027	-1.26678	0.786749
ETF1	0.464872	-1.26893	0.804061
PRDX6	0.64607	-1.27218	0.626109
PTPN1	0.544588	-1.27563	0.731039
TSPO	0.672571	-1.31561	0.643043
