# Supplementary figures and images for: Complement membrane attack complex is an immunometabolic regulator of NLRP3 activation and IL-18 secretion in human macrophages
Source: Front Immunol. 2022 Sep 27;13:918551. doi: 10.3389/fimmu.2022.918551 (PMC9554752; doi:10.3389/fimmu.2022.918551)

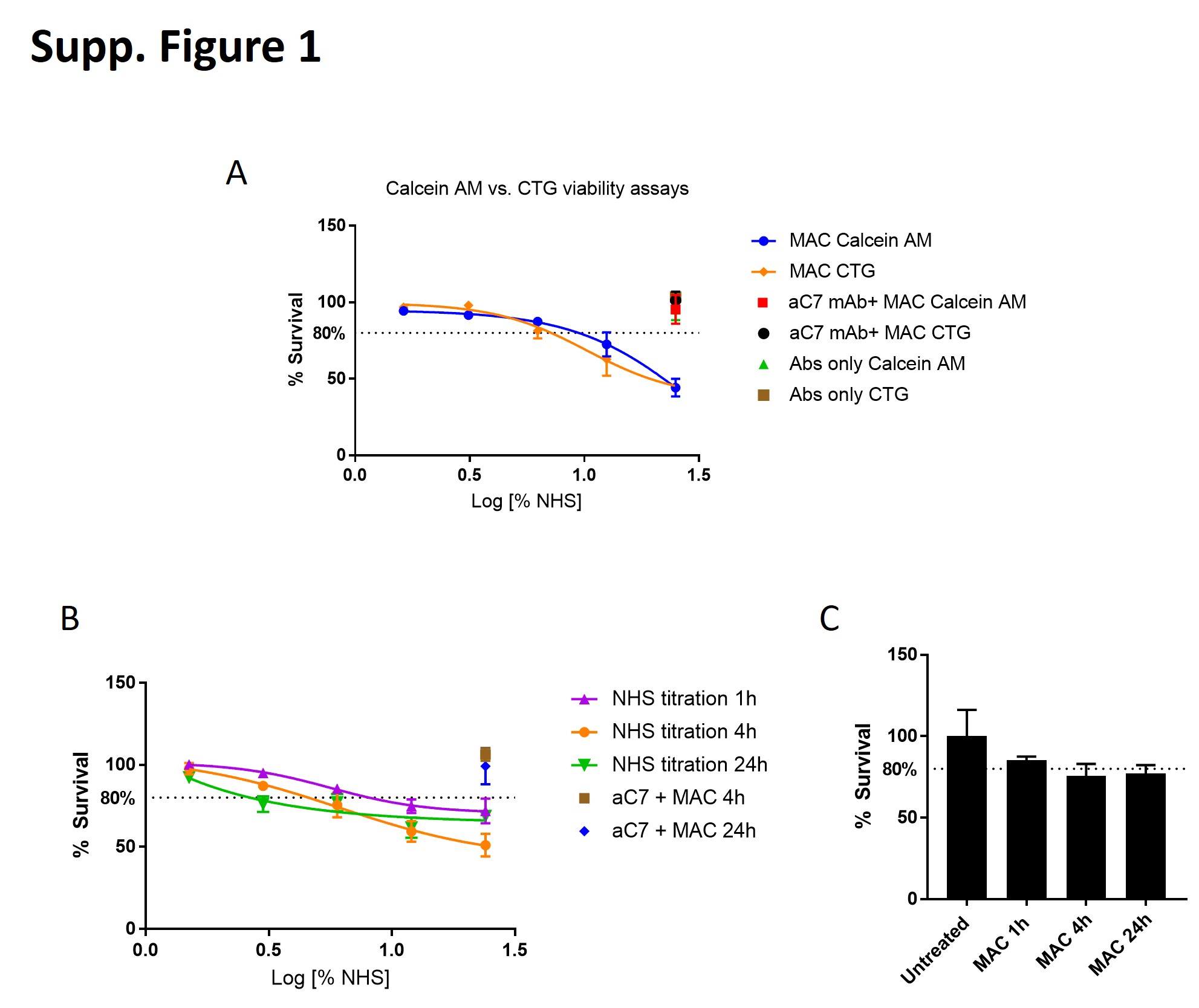

Supplement: Supplementary Figure 1 — (A) CellTiter-Glo and Calcein AM assays to determine hMDMs viability upon antibodies (anti-CD55, CD59, HLA) sensitisation and stimulation with increasing concentrations of NHS for 1 hour. n=3. (B) CellTiter-Glo assay to determine hMDMs viability upon antibodies (anti-CD55, CD59, HLA) sensitisation and stimulation with increasing concentrations of NHS for 1, 4 and 24 hours. (C) Sublytic levels of NHS (6% NHS) at 1 hour (corresponding to 80% survival) 4 and 24 hours (corresponding to 70-80% survival) (n=3). Error bars represent mean +/- S. E. M. [file Image_1.jpeg]

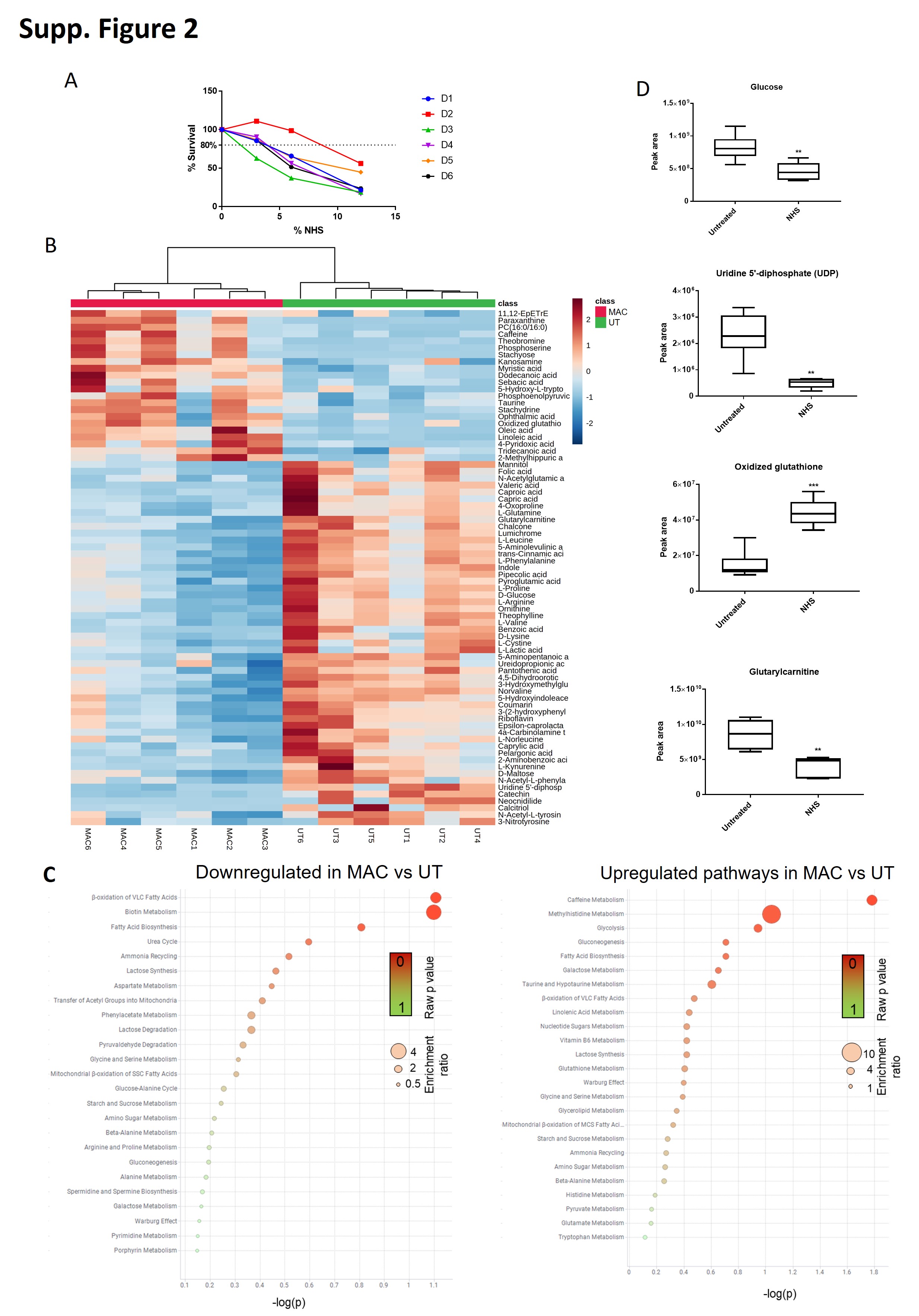

Supplement: Supplementary Figure 2 — (A) Cell viability measurement by CellTiter-Glo or Calcein AM from hMDMs treated with antibodies (anti-CD55, CD59, HLA) and increasing concentrations of NHS for 1 hour, to stablish sublytic complement NHS concentration (80% survival) for each donor (n=6 independent donors). (B) Heat map resulting from targeted metabolomics analysis comparing NHS (named as MAC here) between Untreated conditions for each donor (e. g. Untreated 1-6 referring to each donor labelled). (C) Enrichment pathway analysis using p value and enrichment ratio to rate the most upregulated (right) and downregulated (left) metabolic pathways vs Untreated control. (D) Raw values of peak area from selected metabolites (n=6). Statistical significance in (D) was assessed by unpaired student’s t-test with Welch’s correction for unequal SDs. Error bars represent +/- S. E. M [file Image_2.jpeg]

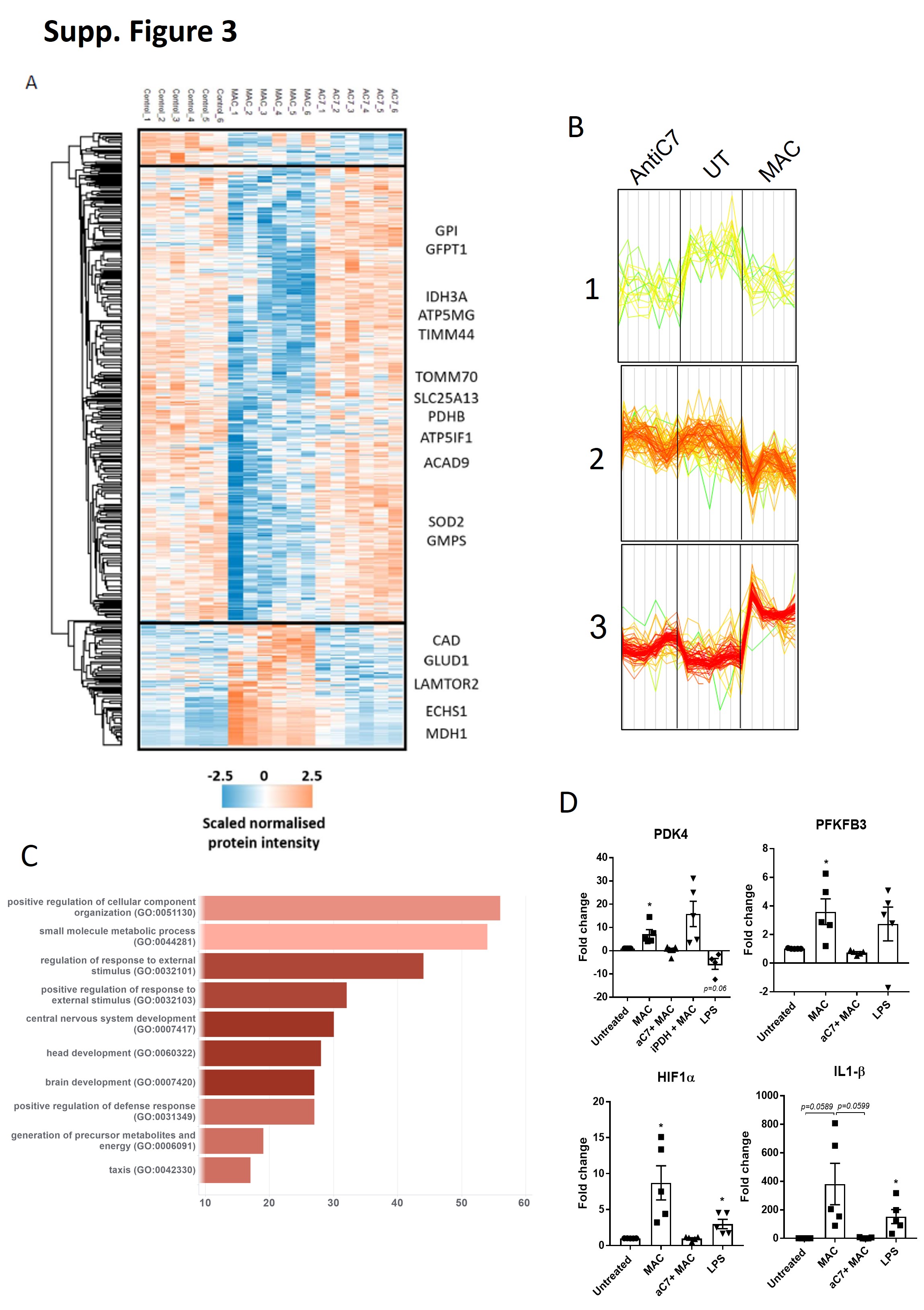

Supplement: Supplementary Figure 3 — Proteomics analysis downstream of sublytic complement attack on hMDMs. (A) Hierarchical clustering of normalized protein intensities (z-score) for significantly regulated proteins (ANOVA permutation=based FDR < 0.05) (N= 724 regulated proteins). Mitochondrial proteins of interest are highlighted in their corresponding cluster. (B) Expression profiles of proteins in three selected clusters corresponding to Supplementary Figure 5A showing distinct behaviours: 1) downregulation in MAC stimulated and anti-C7 stimulated samples; 2) downregulation in MAC treated samples and rescue to UT baseline with anti-C7; and 3) downregulation in MAC treated samples and recovery with anti-C7. (C) Top 10 list of MAC downregulated pathways from proteomics data using statistical enrichment test for GO biological processes analysis (N= 363 downregulated proteins). See methods section. (D) hMDMS treated with MAC (+/- anti-C7) or LPS for 24 hours. Gene expression of HIF-1a, IL-1B, PFKFB3 and PDK4 (iPDH; inhibitor of PDH) normalised to β-actin, HPRT and TBP and expressed as fold change relative to unstimulated hMDMs (n=5). [file Image_3.jpeg]

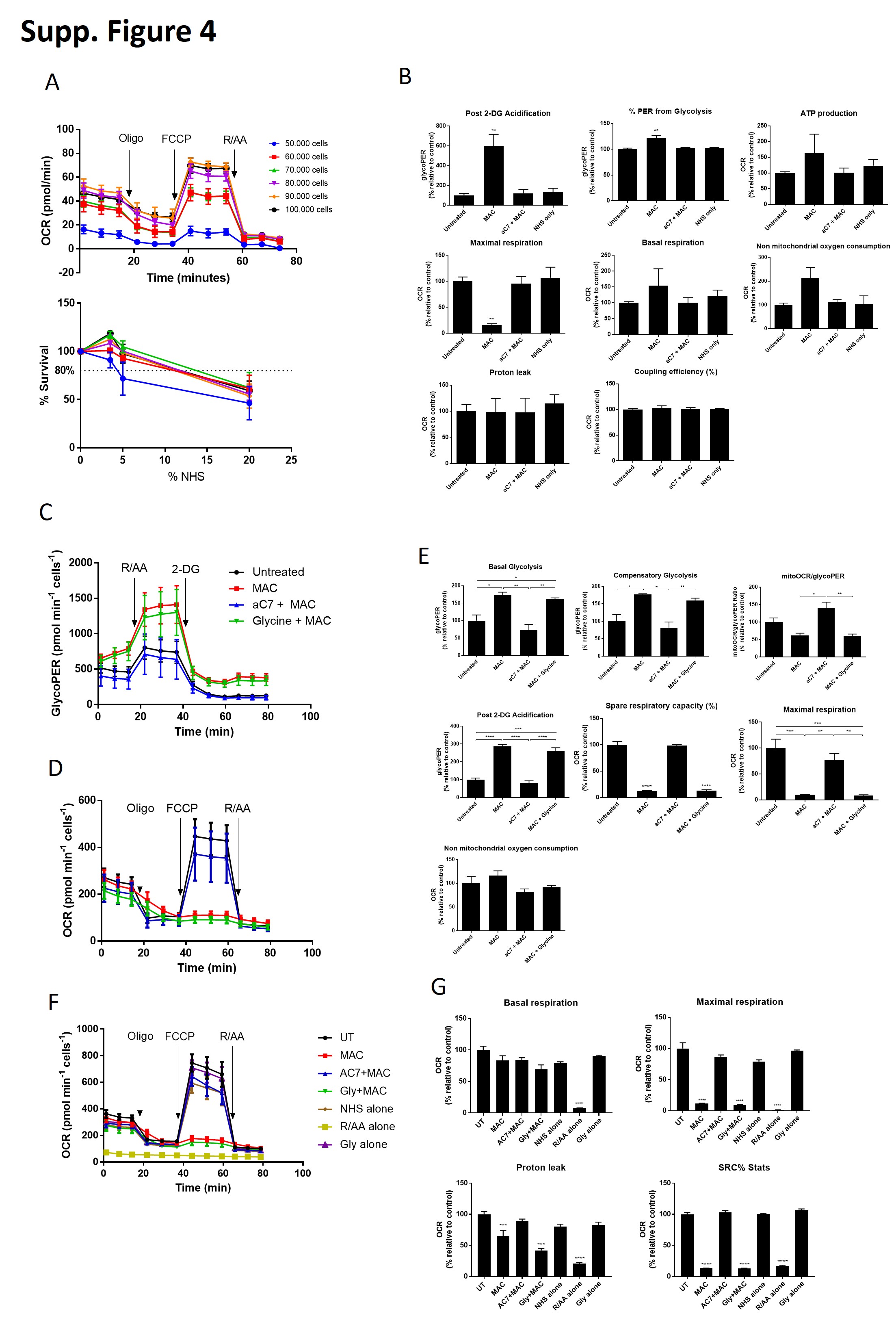

Supplement: Supplementary Figure 4 — (A) hMDMs were differentiated in Cell-Tak coated 96XF seahorse plates at 6 different cell densities. Oxygen consumption rate (OCR) was measured under the Mitochondrial Stress Test in XF96 Seahorse. Cell survival was also measured using Celltiter-Glo assay upon increasing doses of NHS to determine sublytic MAC concentration of NHS at 80% survival in seahorse plates (n=3). (B) Parameters from the seahorse Glycolytic rate test shown in Fig. 3A and the Mito stress test shown in Fig. 3B (n=4). (C–E) hMDMs stimulated with MAC, anti-C7 + MAC or 5 mM Glycine pre-incubated for 50 min plus MAC for 1 hour. (F, G) hMDMs stimulated with MAC, anti-C7 + MAC or 5 mM Glycine pre-incubated for 50 min plus MAC for 24 hour before Mitochondrial Stress Test by Seahorse XF. 0. 5 µM rotenone/antimycin A was added prior to start of test in R/AA condition. Parameters are presented in G. Seahorse measurements: GlycoPER from glycolytic rate test (C) (n=3) and mito stress test (D, F) (n=4), respectively, were measured, and parameters presented in (E, G). Statistical analysis in (B, D, G) was assessed by 1-way ANOVA with post-hoc Tukey’s test. Error bars represent ± S. E. M. [file Image_4.jpeg]

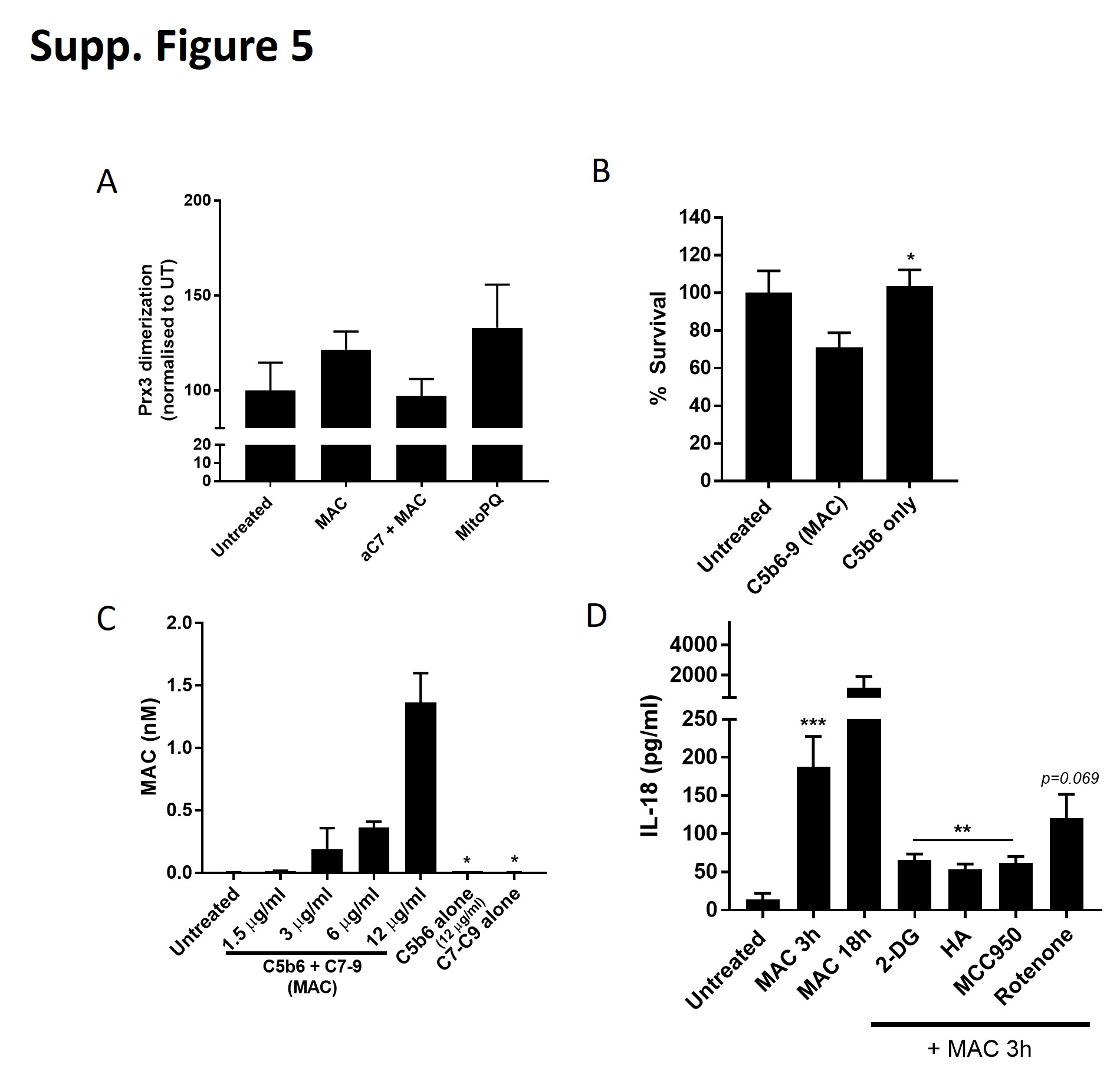

Supplement: Supplementary Figure 5 — (A) WB quantification from , Prx3 dimerization measured by Image J and normalised to untreated (n=3). (B, C) Cells sensitised with neutralising anti-CD59 antibody and stimulated with purified components of MAC. (B) 6 µg/ml of C5b6 plus one molar excess of C7, C8 and C9, viability was measured by CellTiter-Glo (n=4). (C) MAC deposition after set up as in (B) measured by TCC MSD in cell lysates (n=3). (D) IL-18 release by hMDMs detected by IL-18 ELISA of supernatants of hMDMs in 1 million cells per condition (24-well plates) (same 3 donors from which contains additional positive and negative controls) stimulated with MAC for 3 hours with inhibitors 2-DG (5mM), HA (10 μM), and rotenone (0. 5 μM). Statistical analysis performed on (B–D) was assessed by unpaired student’s t-test with Welch’s correction for unequal SDs. Error bars represent ± S. E. M. [file Image_5.jpeg]
